# Supplementary material for: Potential impacts of general practitioners working in or alongside emergency departments in England: initial qualitative findings from a national mixed-methods evaluation
Source: BMJ Open. 2021 May 24;11(5):e045453. doi: 10.1136/bmjopen-2020-045453 (PMC8149439; doi:10.1136/bmjopen-2020-045453)
Supplement: Supplementary data [file bmjopen-2020-045453supp001.pdf]

GPED: System Leader Interviews

What is your current role and what has your role been regarding introduction of GPs into EDs?

Tell us the background to the concept of introducing GPs into EDs as you see it
• Who have been key stakeholders in the idea
• What do they hope to achieve
• Where did it originally come from
• How it fits with other services e.g. walk in centres, 111, out of hours GP
• Have lessons learnt from the experience of introducing other services been incorporated

What is your sense of the 'buy in' from GPs?
• Sustainability (lack of GPs)
• Desirable role for GPs
• What are the challenges/benefits for GPs in this role
• Terms and conditions (e.g. employer, indemnity)

Why do you think the government have decided to invest in GPED?

Describe the different models of GPED care that you are aware of having been/going to be implemented
• Have you got a sense of which might work better (according to what outcomes)?

What do you think patients think about the idea in general?

What do you think the likely impact will be (do you have evidence for this)?
• On ED care delivery from perspective of ED department
• On primary care delivery across the community
• On patient care
• Have you considered unintended consequences (e.g. it will increase demand)
• On primary care delivery across the community
• On patient care
• Have you considered unintended consequences (e.g. it will increase demand)
