# Supplementary material for: Potential impacts of general practitioners working in or alongside emergency departments in England: initial qualitative findings from a national mixed-methods evaluation
Source: BMJ Open. 2021 May 24;11(5):e045453. doi: 10.1136/bmjopen-2020-045453 (PMC8149439; doi:10.1136/bmjopen-2020-045453)
Supplement: Supplementary data [file bmjopen-2020-045453supp004.pdf]

Setting: Prospective Case Sites

Timing: Before introduction of GPED

Participants: Staff in ED

Personal:

What is your current role in the ED?

Do you have a role in relation to the introduction of GPED? If so what is it?

GPED model:

Tell us what you understand about the GPED model that will be implemented in your department

Do you feel that many of the patients you see are 'inappropriate' for ED and should be in primary care (give examples)?

Are you aware of the background to the decision to introduce GPED:

- What it is hoped that GPED will achieve
- What discussions took place
- What options were considered
- What major factors impacted on decision making (if don't mention might want to prompt on waiting time, cost, numbers)
- Was there (describe) consultation process with staff/patients

How is it different from the model you have in place now (is it clearly distinct)

- Structural/organisational requirements for proposed model
- Training requirements
- Timetable for change (date)
- Knowledge/views on the process for selection of patients to be seen by the GP

What are your thoughts on the decision to fund these models of service delivery?

- Does the idea of GPs in ED make sense in general
- For your department
- Are you aware of other types of GPED models being implemented elsewhere

Do staff have a shared understanding of the purpose of the proposed model of GPED?

- Do staff feel they have had sufficient buy in

GPED Topic Guide prosp_before_EDstaff (v1.0) 13-07-2017

- What are your concerns (if any) regarding implementation
- Do you think there are any potential safety issues
- How supported do you feel by management going into the change

Expected impact:

What are your expectations of the impact of the new service on your own everyday working?

- Clinically (type of patients/presenting conditions)
- Working relationships with other staff (e.g. staff selecting patients to be seen by the GP, the GP staff)
- Administratively/organizationally
- For the service provided to patients

What you think the impact will be to your department on:

- Performance (4 hours, hospital admission rate)
- Resources
- How patients use the ED

What do you think will be the key barriers/facilitators to the introduction of GPEP?

What do you think would be deemed to be successful outcomes?

How do you think patients will respond to the new service (satisfaction, ability to feedback, change in behaviour)?

Any other comments to add about GPED

Scantlebury A, *et al. BMJ Open* 2021; 11:e045453. doi: 10.1136/bmjopen-2020-045453
