# Supplementary material for: Potential impacts of general practitioners working in or alongside emergency departments in England: initial qualitative findings from a national mixed-methods evaluation
Source: BMJ Open. 2021 May 24;11(5):e045453. doi: 10.1136/bmjopen-2020-045453 (PMC8149439; doi:10.1136/bmjopen-2020-045453)
Supplement: Supplementary data [file bmjopen-2020-045453supp005.pdf]

Setting: Prospective Case Sites

Timing: 'Before' introduction of GPED/early in implementation process

Participants: GPs

Personal:

What is your current role in the GPED?

What was your previous (or concurrent) role in primary care?

Did you have a role in relation to the introduction of GPED/how did you become aware of the new service model?

Explore decision around taking the role as GP in ED context

Discussion around who is employer, professional indemnity, clinical supervision/support around clinical decision making in role as GP in ED

GPED model:

Tell us what you understand about the GPED model that is being implemented

Are you aware of the background to the decision to introduce GPED:

- What it is hoped that GPED will achieve
- How the service came about
- Consultation process with CCG/other primary care forums

What are your thoughts on the decision to fund these models of service delivery?

- Does the idea of GPs in ED make sense in general
- Aware of other types of GPED models being implemented elsewhere

Do staff (from GP component of service) have a shared understanding of the purpose of the proposed model of GPED?

- Do staff feel they have had sufficient buy in
- What are your concerns (if any) regarding implementation
- Do you think there are any potential safety issues
- How supported do you feel by management going into the change

GPED Topic Guide prosp_before_GPstaff (v1.0) 13-07-2017

Expected impact:

What are your expectations of the impact of the new service on your own everyday working?

- Clinically (type of patients/presenting conditions)
- Working relationships with other staff (e.g. staff selecting patients to be seen by the GP, the ED staff)
- Administratively/organizationally
- For the service provided to patients

What you think the impact will be to your ED department on:

- Performance (4 hours, hospital admission rate)
- Resources
- How patients use the ED

What do you think will be the key barriers/facilitators to the introduction of GPED?

What do you think would be deemed to be successful outcomes?

How do you think patients will respond to the new service (satisfaction, ability to feedback, change in behaviour)?

Any other comments to add about GPED
