# Supplementary material for: Potential impacts of general practitioners working in or alongside emergency departments in England: initial qualitative findings from a national mixed-methods evaluation
Source: BMJ Open. 2021 May 24;11(5):e045453. doi: 10.1136/bmjopen-2020-045453 (PMC8149439; doi:10.1136/bmjopen-2020-045453)
Supplement: Supplementary data [file bmjopen-2020-045453supp006.pdf]

Setting: Prospective Case Sites

Timing: Before introduction of GPED

Participants: Key informants

Personal:

What is your current role in the ED?

What is your role in relation to the introduction of GPED?

ED context:

What model of working with GPs/primary care operates in your ED currently (if any)?

GPED model:

Tell us about the GPED model you are planning to implement

Can you tell us the background to that decision:

- What you are hoping to achieve
- What discussions took place
- What options were considered
- What major factors impacted on decision making (if don't mention might want to prompt on waiting time, cost, numbers)
- Describe the process of consultation (with external bodies e.g. CCG/with internal staff/with patients (or patient reps)

How is it different from the model you have in place now (is it clearly distinct)

- Structural requirements for proposed model
- Organisational requirements for proposed model
- How will changes (if any) be achieved
- Timetable for change (date)

What are your thoughts on the decision to fund these models of service delivery

- does the idea of GPs in ED make sense in general

Do you think this model makes sense/is the right thing for your department?

Do you think staff value the proposed model of service provision?

GPED Topic Guide prosp_before_KI v1.0 13-07-2017

- Do staff have a shared understanding of the purpose of the proposed model of GPED
- Do staff feel they have had sufficient buy in
- What are the concerns (if any) raised by staff regarding implementation
- Can you foresee any potential safety issues

How will you select patients to be seen by the GP and ensure these are the 'right' patients?

- How will you draw the distinction between GP and ED care

Expected impact:

What do you think the impact will be to your department on:

- Performance (4 hours, hospital admission rate)
- Staff (which staff in particular, in what ways)
- Division of labour
- Interaction between different professional groups
- Resources

What impact do you expect GPED to have on patient care?

- Do you think patients will be satisfied with the model
- Do you have a mechanism to collect and/or respond to feedback from patients

Will staff require additional training before implementation

- Which staff and what training in planned/available

How will you judge the success/impact of the new model of service delivery:

- What data might be available for research purposes
- Mechanism for staff feedback about the intervention
- Can the intervention be adapted on the basis of experience
- Patient outcomes

What impact do you think GPED will have on how the public access ED/primary care services?

- How does it sit with other services including walk-in centres, GP practices

Any other comments to add about GPED

GPED Topic Guide prosp_before_KI v1.0 13-07-2017
