# Supplementary material for: Potential impacts of general practitioners working in or alongside emergency departments in England: initial qualitative findings from a national mixed-methods evaluation
Source: BMJ Open. 2021 May 24;11(5):e045453. doi: 10.1136/bmjopen-2020-045453 (PMC8149439; doi:10.1136/bmjopen-2020-045453)
Supplement: Supplementary data [file bmjopen-2020-045453supp007.pdf]

Setting: Prospective Case Sites

Timing: Before GPED

Participants: Patients

What brought you to the ED on this occasion?

Tell us about what happened after you arrived?

- Who did you see first/what happened next
- Description of triage process

Explore reason behind attendance at ED for this consultation – why did they use ED over other potential services (walk-in centres, GP surgery)

- Knowledge of different ways to access health services and what they consider the 'appropriate' ways to use them

Would their experiences on this visit change their consultation choice in the future?

Explore awareness of increased demand on EDs/government funding made available to increase GPs in EDs

- Do they think GPs in ED good idea in principle
- What impact do they think it might have on reducing pressure on EDs

Briefly describe model being proposed and seek comments on that

- What features would make that a good service for patients
- Can see any advantages/disadvantages
- How might they have felt about seeing a GP on this visit

Any other comments to add about GPED.

GPED Topic Guide prosp_before_patient V1.0 13-7-17
