# Supplementary material for: Potential impacts of general practitioners working in or alongside emergency departments in England: initial qualitative findings from a national mixed-methods evaluation
Source: BMJ Open. 2021 May 24;11(5):e045453. doi: 10.1136/bmjopen-2020-045453 (PMC8149439; doi:10.1136/bmjopen-2020-045453)
Supplement: Author's manuscript [file bmjopen-2020-045453.draft_revisions.pdf]

BMJ Open is committed to open peer review. As part of this commitment we make the peer review history of every article we publish publicly available.

When an article is published we post the peer reviewers' comments and the authors' responses online. We also post the versions of the paper that were used during peer review. These are the versions that the peer review comments apply to.

The versions of the paper that follow are the versions that were submitted during the peer review process. They are not the versions of record or the final published versions. They should not be cited or distributed as the published version of this manuscript.

BMJ Open is an open access journal and the full, final, typeset and author-corrected version of record of the manuscript is available on our site with no access controls, subscription charges or pay-per-view fees (http://bmjopen.bmj.com).

If you have any questions on BMJ Open's open peer review process please email
info.bmjopen@bmj.com

BMJ Open

The Potential Impacts of General Practitioners Working in or Alongside Emergency Departments in England

Journal:	*BMJ Open*
Manuscript ID	bmjopen-2020-045453
Article Type:	Original research
Date Submitted by the Author:	01-Oct-2020
Complete List of Authors:	Scantlebury, Arabella; University of York, York Trials Unit, Department of Health Sciences<br>Brant, Heather; University of the West of England, Faculty of Health and Life Sciences<br>Anderson, Helen; University of York<br>Leggett, Heather; University of York, York Trials Unit<br>Salisbury, Chris; University of Bristol, Academic Unit of Primary Health Care<br>Cowlishaw, Sean; The University of Melbourne, Department of Psychiatry<br>Voss, Sarah; University of the West of England, Health and Life Sciences<br>Benger, Jonathan; The University Hospitals NHS Foundation trust, Academic Department of Emergency care; The University of the West of England, Faculty of Health & Life Sciences<br>Adamson, Joy; University of York
Keywords:	ACCIDENT & EMERGENCY MEDICINE, QUALITATIVE RESEARCH, Health policy < HEALTH SERVICES ADMINISTRATION & MANAGEMENT, PRIMARY CARE
Note: The following files were submitted by the author for peer review, but cannot be converted to PDF. You must view these files (e.g. movies) online.	
ISSM_COREQ_Checklist.pdf~RF5ba2acf6.TMP	

SCHOLARONE™
Manuscripts

I, the Submitting Author has the right to grant and does grant on behalf of all authors of the Work (as defined in the below author licence), an exclusive licence and/or a non-exclusive licence for contributions from authors who are: i) UK Crown employees; ii) where BMJ has agreed a CC-BY licence shall apply, and/or iii) in accordance with the terms applicable for US Federal Government officers or employees acting as part of their official duties; on a worldwide, perpetual, irrevocable, royalty-free basis to BMJ Publishing Group Ltd ("BMJ") its licensees and where the relevant Journal is co-owned by BMJ to the co-owners of the Journal, to publish the Work in this journal and any other BMJ products and to exploit all rights, as set out in our licence.

The Submitting Author accepts and understands that any supply made under these terms is made by BMJ to the Submitting Author unless you are acting as an employee on behalf of your employer or a postgraduate student of an affiliated institution which is paying any applicable article publishing charge ("APC") for Open Access articles. Where the Submitting Author wishes to make the Work available on an Open Access basis (and intends to pay the relevant APC), the terms of reuse of such Open Access shall be governed by a Creative Commons licence – details of these licences and which Creative Commons licence will apply to this Work are set out in our licence referred to above.

Other than as permitted in any relevant BMJ Author's Self Archiving Policies, I confirm this Work has not been accepted for publication elsewhere, is not being considered for publication elsewhere and does not duplicate material already published. I confirm all authors consent to publication of this Work and authorise the granting of this licence.

For peer review only

Title: The Potential Impacts of General Practitioners Working in or Alongside Emergency Departments in England

Arabella Scantlebury[1], Heather Brant[2], Helen Anderson[1], Heather Leggett[1], Christopher Salisbury[3], Sean Cowlishaw[4], Sarah Voss[2], Jonathan Benger[5], Joy Adamson[1]

1. Department of Health Sciences, University of York, York, United Kingdom
2. Faculty of Health and Life Sciences, University of the West of England, Bristol, United Kingdom
3. School of Social and Community Medicine, University of Bristol, United Kingdom
4. Department of Psychiatry, The University of Melbourne, Australia
5. Bristol NHS Clinical Commissioning Group, Bristol, United Kingdom.

Corresponding author: Arabella Scantlebury, York Trials Unit, Department of Health Sciences, University of York, YO10 5DD. Arabella.scantlebury@york.ac.uk

Word count: 4164

For peer review only

ABSTRACT

Objectives: To explore the potential impacts of introducing General Practitioners into Emergency Departments (GPED) from the perspectives of service leaders, health professionals and patients. These 'expectations of impact' can be used to generate hypotheses that will inform future implementations and evaluations of GPED.

Design: Qualitative study consisting of 228 semi-structured interviews.

Setting: 10 acute NHS hospitals and the wider healthcare system in England. Interviews were undertaken face-to-face or via telephone. Data were analysed thematically.

Participants: 124 health professionals and 94 patients and carers. 10 service leaders representing a range of national organisations and government departments across England (e.g. NHS England and Department of Health) were also interviewed.

Results: A range of GPED models are being implemented across the NHS due to different interpretations of national policy and variation in local context. This has resulted in stakeholders and organisations interpreting the aims of GPED differently and anticipating a range of potential impacts. Participants expected GPED to affect the following areas: ED performance indicators; patient outcome and experience; service access; staffing and workforce experience; resources. Across these 'domains of influence' arguments for positive, negative, and no effect of GPED were proposed.

Conclusions: Evaluating whether GPED has been successful will be challenging. However, despite uncertainty surrounding the direction of effect, there was agreement across all stakeholder groups on the areas that GPED would influence. As a result, we propose 8 domains of influence that will inform our subsequent mixed-methods evaluation of GPED.

Trial registration: ISRCTN51780222.

Key words: Primary Care; Accident & Emergency Medicine; qualitative research; health policy.

For peer review only - http://bmjopen.bmj.com/site/about/guidelines.xhtml

ARTICLE SUMMARY

Strengths and limitations of the study

- A unique primary study of 10 NHS case sites provides a detailed understanding of the anticipated effects of a current national policy and will inform a wider mixed methods evaluation of the General Practitioners in Emergency Departments (GPED) study.
- Our analysis uses a large qualitative data set and the views of multiple stakeholders.
- Data is from England only and so may not be generalizable to other healthcare settings.
- Data represents the views of individuals who agreed to take part and so may not be exhaustive.

BACKGROUND

Urgent and emergency care is experiencing increasing demand globally.[1] In 2019 attendances at emergency departments (EDs) in England stood at record levels. 2018-19 saw an increase of 4.4% compared with 2017-18, and 21% since 2009-2010.[2]

High levels of ED occupancy lead to crowding,[3] and this can undermine patient safety, clinical outcomes and quality of care,[4-6] delay service delivery,[7] increase associated mortality and reduce patient and clinician satisfaction [8,9]. It has been estimated that between 15% and 40% of patients attending the ED could be treated in general practice.[10-12]

A review of NHS Urgent and Emergency Care in England proposed that selected patients should be directed or "streamed" to an alternative healthcare provider who could better meet their needs, thereby reducing ED attendances.[13] In 2017 this recommendation was translated into policy in the 'Next Steps on the NHS Five Year Forward View' stating that, "Every hospital must have comprehensive front door streaming by October 2017" (p. 15).[14] To provide financial support for the introduction of GPs working in or alongside the ED, the UK government also announced a capital fund of £100million to which hospitals in England could apply.[15-18] Rationales for introducing GPs in or alongside the ED, in addition to addressing the rising demand from perceived primary care patients, have included bringing vital general practice skills and expertise into the ED to improve patient

For peer review only - http://bmjopen.bmj.com/site/about/guidelines.xhtml

care and control costs by reducing admission and investigation rates.[19] What is less clear is how these implicit hypotheses about the effect of GPs in an ED are articulated and understood by policymakers, service leaders, health professionals and patients. These initiatives have not been subject to rigorous, independent evaluation and there is a lack of clarity regarding the assumptions and mechanism(s) through which the predicted performance benefits for these initiatives might be achieved.[20]

In this paper we report findings from qualitative data, which was collected as part of a wider on-going mixed methods study evaluating the impact of GPs working in or alongside the ED (GPED). Further details of the GPED study are outlined in box 1 and in the study protocol.[20] This paper uses qualitative data from service leaders, health professionals and patients to explore the potential impact of introducing GPs into the ED to generate hypotheses that inform how GPED will be evaluated in subsequent research, and implemented into practice.

For peer review only

For peer review only - http://bmjopen.bmj.com/site/about/guidelines.xhtml

METHODS

Design

We completed a qualitative study consisting of interviews with service leaders, health professionals and patients from 10 case study sites (Table 1). The qualitative data reported here was collected as part of the wider GPED study (Box 1), which was approved by East Midlands – Leicester South Research Ethics Committee (ref:17/EM/0312), the University of Newcastle Ethics Committee (Ref: 14348/2016) and also received HRA Approval (IRAS: 230848 and 218038).

Box 1. The GPED Study

Objectives: To evaluate the impact of GPED on patient care, the primary care and acute hospital team and the wider urgent care system.

Design: A mixed methods study consisting of three work packages.

- Work Package A: Mapping, description and classification of current models of GPED in all EDs in England, and interviews with key policymakers to examine the hypotheses that underpin GPED.
- Work Package B: Quantitative analysis of national data to measure the effectiveness, costs and consequences of the GPED models identified in work package A using retrospective analysis of Hospital Episode Statistics.
- Work Package C: detailed mixed methods case studies of different GPED models consisting of: non-participant observation of clinical care, semi-structured interviews with staff, patients and carers, workforce surveys with emergency department staff and analysis of locally available routinely collected hospital data.

PPI: A study PPI group has contributed to research design and materials and data interpretation and dissemination through a series of face-to-face workshops.

Trial status: In progress (ISRCTN51780222)

Funder: National Institute for Health Research (NIHR) Health services and Delivery (HS&DR) Programme.

For peer review only - http://bmjopen.bmj.com/site/about/guidelines.xhtml

Table 1 Data collection

	Service leaders (national)	Case studies (10 hospital sites)
Total number of participants interviewed	10	Health Professionals (124) Patients/carers (94)
Interview type	Semi-structured telephone interviews	Semi-structured face-to-face and telephone interviews
Aim	In-depth understanding from key informants	In-depth understanding from selected case sites
Job roles represented	Department of Health and Social Care, NHS England, NHS Improvement, Royal College of Emergency Medicine	GPs working in the ED, ED doctors (juniors, registrars, consultants), Nurses (streaming, triage, minor injuries, Emergency Nurse Practitioners), ED managerial and clinical leads, clinical directors

For peer review only

Sampling and recruitment

Data were collected from 10 case study sites, which were selected purposively to ensure maximum variation according to: GPED model; GPED duration; geographical location; deprivation index and ED volume (ED attendances). Participants were sampled opportunistically by the research team, whilst undertaking on-site data collection. Service leaders were contacted directly via email.

Data collection

Telephone interviews with service leaders were conducted between December 2017 and January 2018 following informed verbal consent. During interviews participants were asked to describe: their involvement in GPED and background to the policy as well as the expected impact of GPED and any potential unintended consequences (Supplementary material 1).

Case study interviews with patients and health professionals were largely conducted face-to-face at hospital sites during GPED study data collection. Some interviews were conducted

For peer review only - http://bmjopen.bmj.com/site/about/guidelines.xhtml

via telephone at the request of the participant. Written informed consent was provided by all participants and all interviews were audio-recorded. Data collection took place between October 2017 and November 2018 at 10 EDs throughout England. Interviews with health professionals, patients and carers were semi-structured and followed a topic guide (supplementary material 2-7). During interviews health professionals were asked: their current role in ED; details of their GPED model; expected impact. Patients and carers were asked to describe why they chose to attend the ED as well as their experiences. Patients were also asked about their views on introducing GPED and its potential impact.

Analysis

AS, HA, HL and members of the wider GPED research team undertook data collection and analysis. HA is a registered nurse with experience of working in primary care. All other members of the research team involved in data collection and analysis are health services researchers.

Analysis was facilitated by use of the qualitative data management programme NVIVO. After familiarisation, a coding framework was developed through a series of roundtable discussions by the research team and was continually refined and revisited during researcher meetings on an on-going basis throughout data collection and analysis. This framework was used to produce a series of summaries and pen portraits to describe each case site,[21] which informed a final thematic analysis during which themes were refined further for the purpose of this paper.[22] All participants and case sites were allocated unique personal IDs, to protect anonymity and confidentiality. Unless otherwise specified we use the term staff to collectively refer to GP and ED staff throughout the results section.

Patient and Public Involvement

Ten public contributors with experience of using ED services have been directly involved in the design, development and interpretation of the GPED study. In addition to attending external steering group meetings and supporting the development of our original application for research funding and key study materials (e.g information sheets), our ten public contributors have participated in regular workshops throughout the GPED study. During these workshops, public contributors were given copies of anonymised interview transcripts along with pen portraits from two of our study sites. Public contributors initially

For peer review only - http://bmjopen.bmj.com/site/about/guidelines.xhtml

discussed how they interpreted the data, before being asked to consider whether their own interpretations resonated with the research team's framework. Additional workshops are also being held to discuss the wider GPED study's findings where both quantitative and qualitative data will be presented and discussed with the group.

RESULTS

Service leaders and site staff perceived the national implementation of GPED as a response to increasing pressure on EDs, with a lack of supporting research evidence. Many viewed GPED as a top-down, generalised strategy that had been imposed on them without consideration of local context. Ultimately, variations in local context, ED demand and existing GP services in or alongside the ED meant it was not considered possible to implement the same system everywhere. This resulted in a "proliferation of different models", which in turn implied that the impact of GPED on ED performance would vary substantially.

Our qualitative data highlight the challenges associated with a top-down national policy that is implemented in different ways according to local context. We hope to demonstrate the complexity and uncertainty this brings when trying to predict and then evaluate how the policy may impact patients, Emergency Departments and the wider urgent care system. Our results are therefore presented as a series of areas that stakeholders believed would be affected by the introduction of GPED, and the direction of the anticipated effect.

Performance indicators

The premise that ED staff and GPs have inherently different approaches to risk was central to the concept of GPED. GPs were perceived to frame health and illness in a different way to ED staff, with the 'wait and see' culture of primary care leading many to view GPs as more 'risk tolerant' and more appropriately qualified to care for lower acuity patients than their 'risk averse' ED colleagues. This in turn was thought to be beneficial for GPED by making GPs less likely to order unnecessary investigations, or admit or refer lower acuity patients unnecessarily, thereby reducing the time spent in the ED and enhancing patient flow. Despite this general articulation of potential performance benefits, there was significant uncertainty about the impact of GPED within the local systems included in our case studies. One of the main areas of disagreement among site staff and service leaders

For peer review only - http://bmjopen.bmj.com/site/about/guidelines.xhtml

was whether GPs were more tolerant of risk and if so whether this would have adverse consequences for patient safety. This resulted in variation in GPED models across sites. Individual views largely varied according to the degree of integration and the specific role of GPs within the system – making it difficult to identify generalised predictions relating to the potential impact of GPED.

Use of investigations

Many participants were accepting of models that asked GPs to work in a hybrid ED-GP role and encouraged GPs to 'go native', becoming highly integrated within ED teams. Some models were based on the premise that GP access to investigations was crucial to GPED effectiveness – with concerns that the potential scope of GPED would be limited by GPs not being able to undertake investigations and refer to specialties. In contrast, other GPED models limited GPs to working as they would in the community, and service leaders felt strongly that for the model to run effectively GPs and the ED should work separately. There was an idea that GPs 'going native' would encourage them to behave in a similar way to ED doctors, thereby negating any assumed benefits from GPs' different attitudes to risk, investigation and referral. Therefore, prior expectations relating to unnecessary testing were mostly factored into the GPED model at the outset.

Hospital admissions and the 4-hour target

Reducing hospital admissions and improving performance against the 'four-hour standard' (that 95% of ED patients should be discharged, admitted or transferred within 4 hours of arrival) were often quoted as among the potential benefits of GPED. However, this was not universally accepted. For example, some felt that admissions would not be affected, because the population being targeted are not those that would normally be admitted from the ED. Equally, targeting primary care patients was welcomed by ED managers, as although GP patients can be dealt with quickly in theory, in many localities these patients are present in high volumes and were perceived to be at risk of breaching the four-hour standard. However, some feared there might be an unintended worsening effect – diverting people with minor conditions that are theoretically quick to resolve increases the acuity of the remaining ED patient workload. If the ED is left with only high acuity patients, there is a

For peer review only - http://bmjopen.bmj.com/site/about/guidelines.xhtml

possibility that both the time spent in the ED and the proportion of patients who are admitted will increase, worsening the reported "four hour" performance.

When stakeholders discussed possible effects of GPED on performance indicators it was not always clear, and was not model dependent, whether GPED streamed patients were to be included or excluded from the ED figures, and assumptions regarding this influenced participants' views. Generally, performance indicators were considered blunt tools with which to evaluate impact, reflecting potential measurement issues and artefacts rather than good clinical practice. It was also anticipated that the 'visibility' and impact of GPED would be obscured by a year-on-year increase in patient attendances and hospital admissions.

Table 2 Arguments proposed for the potential impact of GPED on ED performance

ED performance and performance indicators				
Potential impact	Positive	Negative	No difference	Exemplar quote
Use of investigations/ testing	Risk tolerant nature of GPs makes them suitable for working alongside the ED – less likely to order investigations unnecessarily	GPs lack skills to work in ED<br><br>By 'going native' and having access to investigations/testing GPs may lose their unique skills and work similarly to ED doctors	Whether GPs were given access to investigations varied depending on the GPED model in place and so any impacts associated with this would be negligible.	*"It was suggested that those problems could be better dealt with by primary care clinicians who had the appropriate skills for the job and would be perhaps confident about seeing and treating and discharging without over-investigation." (Rowan. Staff interview, 07)*
Admissions	Avoid unnecessary admissions of lower acuity patients and improve patient flow	If the ED is left with only high acuity patients the proportion of ED attendances who are admitted will increase	Admissions not affected as the population targeted is not those that would be admitted from ED.	*"But I can't pretend that I think it will make a massive difference on admissions, because the people who are waiting for admission are very largely a different group*

For peer review only - http://bmjopen.bmj.com/site/about/guidelines.xhtml

				of people you see." (Service Leader interview, 02)
Waiting time/ 4 hour KPI	Streaming primary care patients to GP (the most appropriate clinician), reduces the risk of breaching the four hour target as lower acuity patients are high in volume and occupy a lot of clinician time	Diverting patients with minor conditions who are theoretically quick to resolve will increase the acuity of ED work and make improvements in the "4 hour target" less likely. Higher acuity patients are considered more complex and so take longer to manage, increasing the potential for breaching the target	Number of minor breaches that would need to be converted is too large to see any improvement in "4 hour performance"	*"In theory, if you've taken all the minors, all the sort of streamed patients and minor cases out, you'll have ... your staff that are there will be able to devote more time dealing with the majors. And similarly they were hoping that you'd be reducing the volume of patients coming through there but you would hopefully be able to increase the rate the patients were seen. So you would reduce the number of breach patients coming through the main ED department." (Service Leader interview, 07)*

Patient outcome and experience

A process of front door "streaming" of patients on arrival at the ED was intended to match patients with the skill set of the treating clinician. EDs were therefore expected to see improvements in patient outcomes (some of which are reflected in the performance standards) and experience. Streaming lower acuity patients to a GP was anticipated to improve patient care by enabling ED staff to focus on higher acuity patients and ensure that

For peer review only - http://bmjopen.bmj.com/site/about/guidelines.xhtml

GP acuity patients are treated in GPED rather than being ‘sent round the houses’. Patients were aware of the significant resourcing and financial pressures placed on the NHS and so saw value in placing GPs in the ED.

There were concerns, however from service leaders and ED staff, that patient flow could be negatively affected by GPED with a backlog created by patients being required to disclose clinical information on multiple occasions before seeing a GP, or that GPED patients would prevent those with higher acuity needs being seen in a timely manner due to beliefs that GPED may increase the number of patients attending ED and associated crowding (see below).

There was strong and divided opinion between staff groups and even service leaders as to what is considered a ‘GPED appropriate’ patient. Certain assumptions were made about the skill set of GPs, which influenced these views. In some cases, GPs were perceived to lack the appropriate skills and experience to work in the ED, which in turn was felt to limit the potential effectiveness of GPED. Models that required GPs to ‘go native’ were thought to ask GPs to work beyond their clinical competency, with some staff claiming that GPs are not up to date with ED knowledge, and lacking in key clinical skills such as x-ray interpretation and suturing. There were also concerns that GPs may not recognise higher acuity patients, with associated risks to patient safety.

Table 3 Arguments proposed for the potential impact of GPED on patient outcome/experience

Patient outcome and Experience				
Potential impact	Positive	Negative	No difference	Exemplar quote
Streaming patients to the appropriate clinician	Improved flow of patients through the system	Backlog created by patients having to disclose information on multiple occasions before seeing GP.	Annual growth of ED workload may mask impact of GPED on performance	*“Intended impact was to divert as many patients who were able to be streamed to a primary care service, away from the A&E and ED departments, reducing then, surge of patients through and ensuring that patients could be seen quickly and effectively both in A&E and ED, but also in the located primary care services.”(Service Leader interview, 10)*
Patient experience	Improved patient experience by streaming patients to a GP	GPED patients may prevent those with higher acuity being seen		*“I'd like to think if it was working out as we'd originally envisaged that trusts would be able to flow*

For peer review only - http://bmjopen.bmj.com/site/about/guidelines.xhtml

	since this avoids them being 'sent around the houses' and/or waiting in lengthy ED queues, enabling quicker assessment and discharge.	in a timely manner – GPED may increase the number of patients attending ED		*people through the main ED departments much quicker. So we would see reduced breaches. So the four-hour performance would improve but similarly patient experience would significantly improve because you would hopefully be reducing the number of delays to patients getting treated. So hopefully it would just be freeing up the ED department, by taking the streamed patients out. So that's what I was hoping we would see.' (Service Leader interview 07)*
Value of GP	Patients saw value in GPED due to resourcing and financial pressures on NHS	GPs lack appropriate skills and experience to work in ED.		*"What's nice is it takes the pressure off the, er, general A&E and actually emergencies can get deal with emergencies and not get clogged up." (Teak. Patient interview, 021)*

Service Access

There was divided opinion as to how GPED may affect ED attendance. Despite one of the aims of GPED being to create a more efficient service, both staff and patients were concerned that GPED may become a product of its own success by encouraging people to attend ED with primary care problems repeatedly, and that GPED would become a replacement GP service. It was felt that despite any 'educational' component, whereby patients are encouraged to use their own GP when attending GPED, the fact that GPED guaranteed same day access to a GP was in conflict with this message, and could encourage 'inappropriate' attendance with routine rather than urgent care needs. Concerns that GPED could create additional demand on the ED were supported by anecdotal reports from established GPED models highlighting that the volume of patients had increased since introduction. This rise was attributed to the service generating new demand from primary care patients. Others highlighted the potential influence of general practice opening times; because primary care patients tend to present out of hours, GPED could cause peaks in ED attendance when general practice surgeries are closed.

For peer review only - http://bmjopen.bmj.com/site/about/guidelines.xhtml

Yet this view was not universal, service leaders provided various reasons why the policy was unlikely to cause an increase in ED attendance. For example, service leaders argued that given the average person attends the ED less than once a year, it is unlikely that they would start using ED as their main access to general practice. Additionally, as many ED patients present with higher acuity, GPED was not expected to be a supply driver in the same way as a walk-in centre. To this end, GPED was not viewed as being about access to GPs, but about streaming patients to the most clinically appropriate professional. A lack of advertising, the fact that most cases would still be treated in the ED and a lack of patient awareness of GPED was also perceived to mean that GPED would have a negligible impact on demand.

Table 3 Arguments proposed for the potential impact of GPED on ED attendance

Service Access				
Potential impact	Increase	Decrease	No difference	Quote(s)
GPED as a replacement primary care service	GPED becomes a replacement GP service		Streaming patients to most appropriate professional<br><br>Average person uses ED less than once a year so unlikely to become the main source of general practice	*"I guess my personal view is I think they're probably putting GPs on hospitals because they've realised people are fed up of waiting to get an appointment at the GPs and they're going to hospitals, so they're not really fixing the problem there." (Redwood. Patient interview, 02)*
Increase 'inappropriate' attendance	Same day access to a GP may encourage 'inappropriate' attendance	Many patients present with high acuity needs, so not the same as a walk-in centre in terms of supply.		*"But I think, I think what it, what it does do is that, it further reinforces the concept if you've got an urgent and emergency care problem you go to ED, because not only is the ED and x-rays and prescriptions there and all the rest of it there, but now you've got primary care there as*

For peer review only - http://bmjopen.bmj.com/site/about/guidelines.xhtml

				well….I kind of think it acts as a supply site driver." (Service Leader interview, 005)
Increase demand on ED	Peaks in attendance when general practice surgeries are closed.		Patients unaware of GPED service	*"It hasn't been well publicised … patients, I don't think most patients will be aware of it. I think that given they get treated in an emergency department they will probably not recognise that there is, that there's a GP service …" (Service Leader interview, 01)*

For peer review only

Staffing and workforce experience

Staffing issues dominated discussions about the potential impact of GPED, and were seen to pose a major threat to its success. Services leaders and site staff expressed concern that GPED could draw GPs away from primary care and cause competition for GP staff. Consequently, GPED was perceived to have the potential to worsen general practice staffing issues, which in turn could increase waits for a GP appointment and further encourage people to attend ED.

GPED was considered an attractive prospect for those GPs seeking portfolio careers and wishing to expand their practice, knowledge and skills. Traditional general practice was seen as a more stressful and less attractive workplace than newer service models. This was due to several pressures including increasing volume and complexity of workload and depleted community and social care provision. There was some debate as to how the flexible hours associated with GPED would impact on job satisfaction. For example, some anticipated that this flexibility would make it easier to fill rotas, whilst others felt that shift working goes against one of the main reasons why people choose to be a GP.

Table 4 Arguments proposed for the potential impact of GPED on staffing and experience

Staffing and workforce Experience

For peer review only - http://bmjopen.bmj.com/site/about/guidelines.xhtml

Potential impact	Positive	Negative	No effect	Quote(s)
GPs want to work 'beyond the walls of the surgery'	GPED is an attractive place to work for those wanting portfolio careers.	Working 'beyond the walls of the surgery' is not appealing to all and may cause competition for GP staff between primary and secondary care		*"A concern [is] that it would, it would spread the primary care resource more thinly, so it would be less able to respond to, you know, would be less able to respond to sagittal primary care demand…"(Service Leader interview, 05)*
Flexible working hours	Flexible working hours may make it easier to fill rotas	Working out of hours is a deterrent for those who chose to work in general practice		*"Just because I'm a locum I can avoid doing nights, and chose not to do nights." (Chestnut. Staff interview, 22)*
Locum working	Working on a locum or ad hoc basis can be attractive to some and may mitigate against GP staffing issues.	Difficult to ensure the quality of locum staff and inconsistent workforce supply negatively affects collaborative working between ED and GPs		*"The barriers, yes. Often, the GPs are not there all the time, it's not the same person. They're often locum. So, the GP will, sort of, arrive, go straight into their room and then stay in the room unless you call them out for huddle ….. whereas A&E nurses and all of our doctors are all quite social, we're a team, we're really visible to each other. I think just the mentality of a GP is you sit in your room all day, don't you, on your own?" (Nutmeg. Staff interview, 15)*

Many staff perceived GPED to have training and educational benefits for junior doctors who would, in some models, become more confident about discharging patients and build up their primary care knowledge. Conversely, diverting patients with minor conditions to GPED was seen to have benefits for ED juniors and trainees by exposing them to more acutely ill patients.

However, there was a perceived lack of suitably qualified GPs with the necessary skills and experience to work effectively in GPED. Site staff placed importance on making GPED an attractive place to work and ensuring that GPs feel valued, supported and appropriately remunerated for effective implementation. Emphasis was also placed on ensuring GPs feel protected and supported to work within their scope of practice. As a result, some felt that GPs needed to be upskilled or would require extra training. To compensate for this, some respondents emphasised the importance of recruiting experienced GPs, who had previously worked in the ED, or employing GPs that were trained at their hospital site as juniors.

For peer review only - http://bmjopen.bmj.com/site/about/guidelines.xhtml

There was also concern that experienced nursing staff may prefer to work in GPED due to 'better' working hours and it being perceived as an easier job. This not only has implications on ED staffing, but on streaming, which many felt should be undertaken by an experienced nurse. However, some nurses perceived streaming to be a waste of their clinical skills and believed that it took them away from their central role and left ED short-staffed. ED nurse practitioners were also concerned that although they continued to see patients with minor injuries, minor illnesses would be streamed to GPED, which could result in de-skilling of the ED nursing workforce.

Table 5 Arguments proposed for the potential impact embedding GPs in ED teams

	Integrating GPs as part of the ED team		
Potential impact	Positive	Negative	Quote(s)
Training and clinical skills	Benefits for improving team working and skill mix. Training and educational benefits for junior doctors and GPs.	GPs may lack appropriate skills/experience to work in ED	*"Yes, knowledge and experience. GPs could teach about headaches to the primary care nurse and us, if we wanted to help out a little bit, to bring on new nurses who are coming through and learn. Then you could develop majors practitioners, bring them through. Do teaching and education, bring minors and- it would be a perfect bed of opportunity." (Rowan. Staff interview, 20)*
Deskilling of GP and nursing workforce	Nurses prefer to work in GPED	Integrating GPs may cause deskilling.<br><br>Negative views on streaming and the potential for GPED to deskill the nursing workforce by diverting minor illnesses to GPED.	*"There's a risk that the GPs who are then working on a consistent basis within an emergency department or as part of…. that they can go native within that setting and actually take on more of the, qualities that you might expect to see, in other emergency department staff and actually lose the characteristics that*

For peer review only - http://bmjopen.bmj.com/site/about/guidelines.xhtml

			you might expect to see of a GP."(Service Leader interview, 10)

Resources

Staff and patients predicted that GPED would incur higher costs due to the cost of GP employment, and placed importance on ensuring staffing and resources are carefully matched. Staff considered GPs a costly resource and felt that GPs needed to demonstrate their effectiveness. Furthermore, the employment of locums and agency staff to fill these positions was expected to lead to greater costs. There were some concerns that the funding could be better spent improving general practice provision, which may lead to the same outcome. Incidental costs such as paying for training and the set-up and management of new IT systems was considered an added cost and time burden that staff felt had not always been taken into consideration.

Positively, GPED was seen by some as a cost-effective initiative through its presumed effect of reducing hospital admissions and unnecessary patient investigations. If patients were seen by a GP this would release ED staff to treat more unwell patients with a potential cost saving arising from the more effective use of staff resources (i.e. patients being seen by the most appropriate staff member).

Table 6 Arguments proposed for the potential impact of GPED on resources

	Resources		
Potential impact	Positive	Negative	Quote(s)
Costs	Reduction in hospital admissions and patient investigations.<br>Streaming patients to the appropriate clinician may result in cost-savings through more effective use of staff resources.	GPs are a costly resource.<br>Reliance on locums and agency staff.	*"Costs had a massive factor in it. Staffing, we kind of have to work around the cost. So sometimes it's, painfully, not for how many you should have to be able to run the department, it's how many can we afford to have to run the department safely." (Chestnut. Staff interview. 023)*
Infrastructure		Training and IT set-up and management.	*"The training was, I have to say, on the computer system,*

For peer review only - http://bmjopen.bmj.com/site/about/guidelines.xhtml

			not great. I tried to get some IT training on the system. The IT department said there wasn't any training available, but they'd let me know when there was." (Redwood, Staff interview.007)

DISCUSSION

Main findings

Since the 2017 implementation of "comprehensive front door streaming", supported by capital funding [14-18], a variety of different GPED models have been introduced throughout the NHS. This is in part a response to varying local needs and contexts, and also different interpretations of what GPED means on a practical level. This has resulted in disagreement at an individual, stakeholder and organisational level about the purpose and anticipated benefits and dis-benefits of GPED and a lack of clarity about the impact of introducing GPED on these effects. Indeed, for each domain of influence we present there were, in most cases, arguments for positive, negative and no effects of GPED (tables 2-6).

Despite disagreeing about the 'direction of effect,' stakeholders agreed about which areas of the healthcare system and patient care were most likely to be impacted by GPED. This has enabled us to generate 'domains of influence', which will form the basis of our subsequent mixed methods evaluation of the impact of GPED on patient care, the general practice and acute hospital team and the wider urgent care system during the wider GPED study (Box 2).

Box 2: GPED Domains of influence

- Performance against the four hour target/waiting time
- Use of investigations
- Hospital admission
- Patient outcome/experience
- Service access
- Staffing
- Workforce
- Resource use/cost

For peer review only - http://bmjopen.bmj.com/site/about/guidelines.xhtml

Whilst the domains of influence provide the foundation for our wider mixed methods evaluation of GPED, a lack of agreement surrounding the policy's aims, coupled with uncertainty as to how the anticipated impacts will be achieved, poses a significant challenge when evaluating whether GPED can be considered a successful national policy.

It is also unclear whether the success of GPED should be determined by its effect on EDs or the wider healthcare system. This warrants careful consideration since some domains, such as ED costs or performance, may be improved at the expense of the wider NHS. Additionally, many of the differences in opinion surrounding the potential impact of GPED are underpinned by confusion as to whether patients attending the GPED are considered part of, or separate from, the denominator used for measuring ED performance. This has implications for understanding the effect of GPED on key performance indicators, particularly the “4 hour target”.

Comparison with existing literature

In 2010 Carson et al explored rationales for the introduction of GPED through an online survey. They report that “The main reason was to meet the needs of patients or improve quality of care. This was followed by achieving the four-hour target and reducing cost.”[19]

Similar assumptions have persisted, and were seen to be drivers of the policy initiative to roll out GPED in all EDs across England. Benefits of GPED, particularly to address the increasing demand in emergency care, were perpetuated through rhetoric presented in the national press [23], clinical press releases [24], medical journals [17, 25] and within the policy documents produced at the time [26, 27].

Early studies appeared to underpin some of these assumptions. Evaluations of early adopters in the UK and Europe suggested that GPs in the ED could “result in reduced rates of investigations, prescriptions, and referrals”,[28, 29] increase patient satisfaction,[30] and offer patients a greater range of healthcare provision.[31] However, these studies have generally been of poor quality.

For peer review only - http://bmjopen.bmj.com/site/about/guidelines.xhtml

More recently, these assumed benefits have been challenged. A realist review concluded that despite a reduction in process time for non-urgent patients this does not necessarily increase capacity to care for the sickest patients.[32] The main cause of ED crowding is a lack of beds and congestion in the flow of sicker patients rather than absolute attendance numbers.[33] In addition, GPED may encourage patients to present to the ED with a primary care problem, with consequent increases in ED attendance.[34, 35]

To date, reviews that examine GPED in more detail have concluded that there is insufficient evidence to support national policy or local system change.[35-37] Two Cochrane reviews (2012 and 2018) concluded that there was “insufficient evidence upon which to draw conclusions for practice or policy regarding the effectiveness and safety of care provided to non-urgent patients by GPs versus EPs in the ED to mitigate problems of overcrowding, wait-times and patient flow” (p.2).[38, 39]

Strengths and Limitations

The ‘domains of influence’ that we have identified in this paper were generated from a large evaluation that used ‘big qualitative data’ (228 interviews) and the views of multiple stakeholders. This provided a rich and nuanced understanding of the complexity surrounding a current national policy – GPED. Our data apply to England only, and so may not be generalizable to other healthcare settings. In addition, we could only interview those who agreed to take part, and although our data spans a very wide range of individuals and views it is unlikely to be exhaustive. The detail we have obtained has enabled us to propose the domains of influence that will be used to inform our wider GPED study, the aim of which is to evaluate the impact of GPED on each of the domains of influence in detail. It could be argued that the data we present here represents the inherent uncertainty and resistance to change that most healthcare policy encounters prior to or during early implementation, and so is representative of typical ‘teething problems.’ However, while it is assumed that such issues will improve over time, recent research suggests that issues that are identified early in the implementation process often persist long after establishment.[40] It is our hope that by identifying ‘domains of influence’, rather than a set of hypotheses, we have mitigated against this and have identified many of the key areas that the GPED policy is likely to affect, whilst providing a framework to guide our forthcoming mixed methods evaluation.

For peer review only - http://bmjopen.bmj.com/site/about/guidelines.xhtml

CONCLUSION

In 2017, a significant financial commitment to support hospitals introduce GPs in ED was made in a direct attempt to address growing concerns surrounding the pressures on emergency departments. However, the reality of introducing GPs in ED is complex. Throughout the NHS, the policy is being interpreted differently, which has created a range of GPED models to be implemented into ever-changing and variable local contexts. This variation both in terms of how the policy is being interpreted and introduced, different 'baseline levels' of GPED and the lack of agreement from stakeholders surrounding the potential benefits and dis-benefits of the policy, mean that the impact of GPED is difficult to predict. However, our findings suggest that GPED will affect 8 key areas. These 'domains of influence' will be used as the foundation for our subsequent mixed methods evaluation.

ACKNOWLEDGEMENTS

The authors would like to thank the participants for their involvement in the study, the GPED public contributors and wider GPED research team.

FUNDING STATEMENT

This work was supported by the National Institute for Health Research (NIHR) Health Services & Delivery Research (HS&DR) Programme, project number 15/145/06.

DISCLAIMER

The views and opinions expressed therein are those of the authors and do not necessarily reflect those of the HS&DR Research Programme, NIHR, NHS or the Department of Health.

COMPETING INTERESTS

Jonathan Benger is seconded part-time to the post of interim Chief Medical Officer at NHS Digital. All other authors declare no conflict of interest.

ETHICS APPROVAL

Approval for the study has been obtained from the Health Research Authority (HRA) (IRAS: 230848 and 218038). The protocol was reviewed and received a favourable opinion from the NHS East Midlands – Leicester South Research Ethics Committee REC: 17/EM/0312 and the University of Newcastle Ethics Committee (Ref: 14348/2016) a

For peer review only

PROVENANCE AND PEER REVIEW

Not commissioned; peer reviewed for ethics and funding approval prior to submission.

DATA SHARING STATEMENT

The data set which we have acquired will not be available as our ethical approval does not permit the sharing of the entire dataset.

AUTHOR CONTRIBUTIONS

AS drafted the manuscript, undertook data collection and analysis. HA and HL, undertook data collection and analysis and critically appraised the manuscript. HB, CS and SV critically appraised the manuscript. JA and JB helped to draft the manuscript – JA also undertook analysis. JA, JB, CS and SV designed the study. All authors have reviewed and approved the final manuscript.

REFERENCES

1. Berchet, C., *Emergency Care Services: Trends, Drivers and Interventions to Manage the Deman*. 2015, Organisation for Economic Co-operation and Development: Paris, France.
2. NHS Digital, *Hospital Accident & Emergency Activity 2018-19*. 2019, NHS Digital: England.
3. Pines, J.M. and R.T. Griffey, *What We Have Learned From a Decade of ED Crowding Research.* Academic Emergency Medicine, 2015. **22**(8): p. 985-987.
4. Bernstein, S.L., et al., *The Effect of Emergency Department Crowding on Clinically Oriented Outcomes.* Academic Emergency Medicine, 2009. **16**(1): p. 1-10.
5. Collis, J., *Adverse effects of overcrowding on patient experience and care.* Emergency nurse, 2010. **18**(8).
6. Morley, C., et al., *Emergency department crowding: A systematic review of causes, consequences and solutions.* PloS one, 2018. **13**(8): p. e0203316-e0203316.
7. Rasouli, H.R., et al., *Outcomes of Crowding in Emergency Departments; a Systematic Review.* Archives of academic emergency medicine, 2019. **7**(1): p. e52-e52.
8. Richardson, D.B., *Increase in patient mortality at 10 days associated with emergency department overcrowding.* The Medical journal of Australia, 2006. **184**(5): p. 213-216.
9. Higginson, I., *Emergency department crowding.* Emergency Medicine Journal, 2012. **29**(6): p. 437-443.
10. NHS England, *Transforming urgent and emergency care services in England. Urgent and emergency care review end of phase 1 report*, in *High quality care for all, now and for future generations*. 2013, NHS England: London.
11. Murphy, K. and C. Mann, *Time to Act–Urgent Care and A&E: the patient perspective*. 2015, Royal College of Emergency Medicine & The Patients Association: London.
12. Thompson, M.I.W., et al., *Suitability of emergency department attenders to be assessed in primary care: survey of general practitioner agreement in a random sample of triage records analysed in a service evaluation project.* BMJ Open, 2013. **3**(12): p. e003612.
13. Urgent and Emergency Care Review Team and Emergency Care Intensive Support Team, *Transforming urgent and emergency care services in England. Safer, faster, better: good*

For peer review only - http://bmjopen.bmj.com/site/about/guidelines.xhtml

practice in delivering urgent and emergency care. A guide for local health and social care communities*. 2015, NHS England: London.

14. NHS England, *Next Steps on the NHS Five Year Forward View*. 2017: England.
15. Department of Health and Social Care, *Funds for next winter will ease pressure on accident and emergency (A&E) departments in England.* 2017: London.
16. HM Treasury, *Spring Budget 2017*. 2017: London.
17. Iacobucci, G., *All emergency departments must have GP led triage by October.* BMJ, 2017. **356**: p. j1270.
18. Iacobucci, G., *NHS gets £100m budget "sticking plaster" to help manage emergency demand.* BMJ, 2017. **356**: p. j1248.
19. Carson, D., H. Clay, and R. Stern, *Primary care and emergency departments: report from the Primary Care Foundation. Mar 2010*. 2010, Primary Care Foundation: London.
20. Morton, K., et al., *General practitioners and emergency departments (GPED)—efficient models of care: a mixed-methods study protocol.* BMJ Open, 2018. **8**(10): p. e024012.
21. Sheard, L. and C. Marsh, *How to analyse longitudinal data from multiple sources in qualitative health research: the pen portrait analytic technique.* BMC Medical Research Methodology, 2019. **19**(1): p. 169.
22. Braun, V. and V. Clarke, *Using thematic analysis in psychology.* Qualitative Research in Psychology, 2006. **3**(2): p. 77-101.
23. Campbell, D., *NHS chiefs order hospitals to begin urgent overhaul of A&E care*, in *The Guardian*. 2017, The Guardian: London, UK.
24. Royal College of Emergency Medicine and Urgent Health UK, *The Royal College of Emergency Medicine and Urgent Health UK call for the colocation of urgent care services with A&E departments* 2015: RCEM.
25. Wickware, C., *Nearly 420 extra GPs needed across country to deliver A&E streaming service* in *Pulse*. 2017.
26. Stevens, S.a.M., J, *Action to get A&E performance back on track*, A.N.P.T.C. Executives, et al., Editors. 2017, NHS England and NHS Improvement.
27. Department of Health and Social Care., *A&E departments to get more funding*. 2017: GOV.UK
28. Dale, J., et al., *Primary care in the accident and emergency department: II. comparison of general practitioners and hospital doctors.* BMJ, 1995. **311**(7002): p. 427-430.
29. Wang, M., et al., *Hospital-integrated general practice: a promising way to manage walk-in patients in emergency departments.* Journal of Evaluation in Clinical Practice, 2014. **20**(1): p. 20-26.
30. Boeke, A.J.P., et al., *Effectiveness of GPs in accident and emergency departments.* British Journal of General Practice, 2010. **60**(579): p. e378-e384.
31. Bickerton, J., et al., *Streaming primary urgent care: a prospective approach.* Primary Health Care Research & Development, 2012. **13**(2): p. 142-152.
32. Cooper, A., et al., *The impact of general practitioners working in or alongside emergency departments: a rapid realist review.* BMJ Open, 2019. **9**(4): p. e024501.
33. Oliver, D., *David Oliver: Why force GP streaming on NHS emergency departments?* BMJ, 2020. **368**: p. m992.
34. Blunt, I.E., N and Merry, L., *What's Behind the A&E 'Crisis'?* , N. Trust, Editor. 2015.
35. Ramlakhan, S., et al., *Primary care services located with EDs: a review of effectiveness.* Emergency Medicine Journal, 2016. **33**(7): p. 495-503.
36. Fisher, J., B. Salman, and M. Cooke, *A rapid review exploring the interface between primary and emergency care in England.* Primary Care and Emergency Departments: Primary Care Foundation, 2010.

For peer review only - http://bmjopen.bmj.com/site/about/guidelines.xhtml

37. Turner, J., et al., *What evidence is there on the effectiveness of different models of delivering urgent care? A rapid review*. Health Services and Delivery Research. 2015: NIHR Journals Library, Southampton (UK).
38. Khangura, J.K., et al., *Primary care professionals providing non-urgent care in hospital emergency departments.* Cochrane Database of Systematic Reviews, 2012(11).
39. Gonçalves-Bradley, D., et al., *Primary care professionals providing non-urgent care in hospital emergency departments.* Cochrane Database of Systematic Reviews, 2018(2).
40. Anderson, H., Y. Birks, and J. Adamson, *Exploring the relationship between nursing identity and advanced nursing practice: An ethnographic study.* Journal of Clinical Nursing, 2020. **29**(7-8): p. 1195-1208.

For peer review only

For peer review only - http://bmjopen.bmj.com/site/about/guidelines.xhtml

GPED: System Leader Interviews

What is your current role and what has your role been regarding introduction of GPs into EDs?

Tell us the background to the concept of introducing GPs into EDs as you see it
• Who have been key stakeholders in the idea
• What do they hope to achieve
• Where did it originally come from
• How it fits with other services e.g. walk in centres, 111, out of hours GP
• Have lessons learnt from the experience of introducing other services been incorporated

What is your sense of the 'buy in' from GPs?
• Sustainability (lack of GPs)
• Desirable role for GPs
• What are the challenges/benefits for GPs in this role
• Terms and conditions (e.g. employer, indemnity)

Why do you think the government have decided to invest in GPED?

Describe the different models of GPED care that you are aware of having been/going to be implemented
• Have you got a sense of which might work better (according to what outcomes)?

What do you think patients think about the idea in general?

What do you think the likely impact will be (do you have evidence for this)?
• On ED care delivery from perspective of ED department
• On primary care delivery across the community
• On patient care
• Have you considered unintended consequences (e.g. it will increase demand)
• On primary care delivery across the community
• On patient care
• Have you considered unintended consequences (e.g. it will increase demand)

Setting: Established Case Sites

Participants: Staff in ED/GPED/KI

What is your current role in the GPED?

What model of working with GPs/primary care operates in your ED currently?

Were you involved (and in what way) in the design or initial implementation of GPED?

- only if indicate were involved, ask planning/implementation questions

Planning/implementation stage:

What can you tell us about the initial process of design and implementation of this service

- Key staff involved
- Structural/organisational changes
- Decision making/service design
- Consultation with staff/patients/external bodies

What was expected to be achieved by the change?

What were the key barriers/facilitators?

What were the key issues for staff before the introduction?

What was the attitude/approach to change from management?

Impact:

How do you think the GPED model is working?

- Process of selecting patients to be seen by the GP/streaming/getting the 'right' patients
- Key advantages/disadvantages
- Any safety issues

How has it impacted on overall workings of the ED?

- Has there been any impact on performance (e.g. 4 hours, hospital admission rate)
- Resources

Do you think any improvements could be made to the GPED model (aware of different service configurations in other places)?

For peer review only - http://bmjopen.bmj.com/site/about/guidelines.xhtml

What feedback have you had from patients about the GPED model (are they satisfied etc)?

Do you think the availability of this GPED model is likely to change the way the public decide how, where and when to seek care?

For emergency care staff:

How has GPED impacted on your own everyday working?

- Clinically (type of patients/presenting conditions)
- Working relationships with other staff (e.g. the staff who select patients to be seen by GP, the GP staff)
- Service provided to patients
- Administratively/organizationally
- Any surprises

For general practice staff in GPED:

How is care organised within GP component of GPED?

How does practice within GPED compare to other services (GP practice, walk-in centres):

- Clinically (types of patients/presenting conditions)
- Patient 'outcomes' (e.g. referrals, requests for testing, transfer back to ED)
- Interaction with other professional groups within GP component/ED staff
- Workload
- Any surprises

Discussion around who is employer, professional indemnity, clinical supervision/support around clinical decision making in role as GP in ED

Do you feel you act differently as a practitioner following time in ED (probe – both back in primary care and over time within ED)

Satisfaction with role of GP in ED

- Met with expectations
- Plan to continue in role
- Career plans

How do you think patients have responded to the service?

For peer review only - http://bmjopen.bmj.com/site/about/guidelines.xhtml

GPED

- Why they came to AE rather than GP practice
- Satisfaction with GPED

Any other comments to add about GPED

For peer review only

For peer review only - http://bmjopen.bmj.com/site/about/guidelines.xhtml

GP ED

UWE Bristol University of the West of England

Setting: Existing Case Sites

Participants: Patients

What brought you to the ED on this occasion?

Tell us about what happened after you arrived?

- Who did you see first/what happened next
- Description of being selected to be seen by the GP

Did you know it was possible to be sent to a GP after coming to ED?

- Was this communicated to you
- Did you understand the process/reason you were selected for the GP
- How did you feel about being seen by a GP
- Have you any previous experience of this service (give example)

Explore reason behind attendance at ED for this consultation – why did they use ED over other potential services (walk-in centres, GP surgery)

- Knowledge of different ways to access health services and what they consider the ‘appropriate’ ways to use them

Would their experiences on this visit change their consultation choice in the future?

Explore awareness of increased demand on EDs/government funding made available to increase GPs in EDs

- Do they think GPs in ED good idea in principle
- What impact do they think it might have on reducing pressure on EDs
- Do they think it will change what patients do

How does practice within GPED compare to other GP services?

How satisfied are they with the visit?

- How long did you have to wait
- How satisfied are you with the outcome
- Can you think of any ways you could improve the service?
- Opportunity to provide feedback

Any other comments to add about GPED.

For peer review only - http://bmjopen.bmj.com/site/about/guidelines.xhtml

Setting: Prospective Case Sites

Timing: Before introduction of GPED

Participants: Staff in ED

Personal:

What is your current role in the ED?

Do you have a role in relation to the introduction of GPED? If so what is it?

GPED model:

Tell us what you understand about the GPED model that will be implemented in your department

Do you feel that many of the patients you see are 'inappropriate' for ED and should be in primary care (give examples)?

Are you aware of the background to the decision to introduce GPED:

- What it is hoped that GPED will achieve
- What discussions took place
- What options were considered
- What major factors impacted on decision making (if don't mention might want to prompt on waiting time, cost, numbers)
- Was there (describe) consultation process with staff/patients

How is it different from the model you have in place now (is it clearly distinct)

- Structural/organisational requirements for proposed model
- Training requirements
- Timetable for change (date)
- Knowledge/views on the process for selection of patients to be seen by the GP

What are your thoughts on the decision to fund these models of service delivery?

- Does the idea of GPs in ED make sense in general
- For your department
- Are you aware of other types of GPED models being implemented elsewhere

Do staff have a shared understanding of the purpose of the proposed model of GPED?

- Do staff feel they have had sufficient buy in

For peer review only - http://bmjopen.bmj.com/site/about/guidelines.xhtml

- What are your concerns (if any) regarding implementation
- Do you think there are any potential safety issues
- How supported do you feel by management going into the change

Expected impact:

What are your expectations of the impact of the new service on your own everyday working?

- Clinically (type of patients/presenting conditions)
- Working relationships with other staff (e.g. staff selecting patients to be seen by the GP, the GP staff)
- Administratively/organizationally
- For the service provided to patients

What you think the impact will be to your department on:

- Performance (4 hours, hospital admission rate)
- Resources
- How patients use the ED

What do you think will be the key barriers/facilitators to the introduction of GPEP?

What do you think would be deemed to be successful outcomes?

How do you think patients will respond to the new service (satisfaction, ability to feedback, change in behaviour)?

Any other comments to add about GPED

For peer review only - http://bmjopen.bmj.com/site/about/guidelines.xhtml

Setting: Prospective Case Sites

Timing: 'Before' introduction of GPED/early in implementation process

Participants: GPs

Personal:

What is your current role in the GPED?

What was your previous (or concurrent) role in primary care?

Did you have a role in relation to the introduction of GPED/how did you become aware of the new service model?

Explore decision around taking the role as GP in ED context

Discussion around who is employer, professional indemnity, clinical supervision/support around clinical decision making in role as GP in ED

GPED model:

Tell us what you understand about the GPED model that is being implemented

Are you aware of the background to the decision to introduce GPED:

- What it is hoped that GPED will achieve
- How the service came about
- Consultation process with CCG/other primary care forums

What are your thoughts on the decision to fund these models of service delivery?

- Does the idea of GPs in ED make sense in general
- Aware of other types of GPED models being implemented elsewhere

Do staff (from GP component of service) have a shared understanding of the purpose of the proposed model of GPED?

- Do staff feel they have had sufficient buy in
- What are your concerns (if any) regarding implementation
- Do you think there are any potential safety issues
- How supported do you feel by management going into the change

For peer review only - http://bmjopen.bmj.com/site/about/guidelines.xhtml

GP+ED

UWE Bristol University of the West of England

Expected impact:

What are your expectations of the impact of the new service on your own everyday working?

- Clinically (type of patients/presenting conditions)
- Working relationships with other staff (e.g. staff selecting patients to be seen by the GP, the ED staff)
- Administratively/organizationally
- For the service provided to patients

What you think the impact will be to your ED department on:

- Performance (4 hours, hospital admission rate)
- Resources
- How patients use the ED

What do you think will be the key barriers/facilitators to the introduction of GPED?

What do you think would be deemed to be successful outcomes?

How do you think patients will respond to the new service (satisfaction, ability to feedback, change in behaviour)?

Any other comments to add about GPED

For peer review only

For peer review only - http://bmjopen.bmj.com/site/about/guidelines.xhtml

GPED

UWE Bristol University of the West of England

Setting: Prospective Case Sites

Timing: Before introduction of GPED

Participants: Key informants

Personal:

What is your current role in the ED?

What is your role in relation to the introduction of GPED?

ED context:

What model of working with GPs/primary care operates in your ED currently (if any)?

GPED model:

Tell us about the GPED model you are planning to implement

Can you tell us the background to that decision:

- What you are hoping to achieve
- What discussions took place
- What options were considered
- What major factors impacted on decision making (if don't mention might want to prompt on waiting time, cost, numbers)
- Describe the process of consultation (with external bodies e.g. CCG/with internal staff/with patients (or patient reps)

How is it different from the model you have in place now (is it clearly distinct)

- Structural requirements for proposed model
- Organisational requirements for proposed model
- How will changes (if any) be achieved
- Timetable for change (date)

What are your thoughts on the decision to fund these models of service delivery

- does the idea of GPs in ED make sense in general

Do you think this model makes sense/is the right thing for your department?

Do you think staff value the proposed model of service provision?

For peer review only - http://bmjopen.bmj.com/site/about/guidelines.xhtml

- Do staff have a shared understanding of the purpose of the proposed model of GPED
- Do staff feel they have had sufficient buy in
- What are the concerns (if any) raised by staff regarding implementation
- Can you foresee any potential safety issues

How will you select patients to be seen by the GP and ensure these are the 'right' patients?

- How will you draw the distinction between GP and ED care

Expected impact:

What do you think the impact will be to your department on:

- Performance (4 hours, hospital admission rate)
- Staff (which staff in particular, in what ways)
- Division of labour
- Interaction between different professional groups
- Resources

What impact do you expect GPED to have on patient care?

- Do you think patients will be satisfied with the model
- Do you have a mechanism to collect and/or respond to feedback from patients

Will staff require additional training before implementation

- Which staff and what training in planned/available

How will you judge the success/impact of the new model of service delivery:

- What data might be available for research purposes
- Mechanism for staff feedback about the intervention
- Can the intervention be adapted on the basis of experience
- Patient outcomes

What impact do you think GPED will have on how the public access ED/primary care services?

- How does it sit with other services including walk-in centres, GP practices

Any other comments to add about GPED

For peer review only - http://bmjopen.bmj.com/site/about/guidelines.xhtml

Setting: Prospective Case Sites

Timing: Before GPED

Participants: Patients

What brought you to the ED on this occasion?

Tell us about what happened after you arrived?

- Who did you see first/what happened next
- Description of triage process

Explore reason behind attendance at ED for this consultation – why did they use ED over other potential services (walk-in centres, GP surgery)

- Knowledge of different ways to access health services and what they consider the 'appropriate' ways to use them

Would their experiences on this visit change their consultation choice in the future?

Explore awareness of increased demand on EDs/government funding made available to increase GPs in EDs

- Do they think GPs in ED good idea in principle
- What impact do they think it might have on reducing pressure on EDs

Briefly describe model being proposed and seek comments on that

- What features would make that a good service for patients
- Can see any advantages/disadvantages
- How might they have felt about seeing a GP on this visit

Any other comments to add about GPED.

For peer review only - http://bmjopen.bmj.com/site/about/guidelines.xhtml

BMJ Open

The Potential Impacts of General Practitioners Working in or Alongside Emergency Departments in England: initial qualitative findings from a national mixed-methods evaluation

Journal:	*BMJ Open*
Manuscript ID	bmjopen-2020-045453.R1
Article Type:	Original research
Date Submitted by the Author:	17-Feb-2021
Complete List of Authors:	Scantlebury, Arabella; University of York, York Trials Unit, Department of Health Sciences<br>Brant, Heather; University of the West of England, Faculty of Health and Life Sciences<br>Anderson, Helen; University of York<br>Leggett, Heather; University of York, York Trials Unit<br>Salisbury, Chris; University of Bristol, Academic Unit of Primary Health Care<br>Cowlishaw, Sean; The University of Melbourne, Department of Psychiatry<br>Voss, Sarah; University of the West of England, Health and Life Sciences<br>Benger, Jonathan; NHS Bristol North Somerset and South Gloucestershire Clinical Commissioning Group, Academic Department of Emergency care; University of the West of England, Faculty of Health & Life Sciences<br>Adamson, Joy; University of York
**Primary Subject Heading**:	Emergency medicine
Secondary Subject Heading:	Health services research, Health policy, Qualitative research, Emergency medicine
Keywords:	ACCIDENT & EMERGENCY MEDICINE, QUALITATIVE RESEARCH, Health policy < HEALTH SERVICES ADMINISTRATION & MANAGEMENT, PRIMARY CARE

SCHOLARONE™
Manuscripts

I, the Submitting Author has the right to grant and does grant on behalf of all authors of the Work (as defined in the below author licence), an exclusive licence and/or a non-exclusive licence for contributions from authors who are: i) UK Crown employees; ii) where BMJ has agreed a CC-BY licence shall apply, and/or iii) in accordance with the terms applicable for US Federal Government officers or employees acting as part of their official duties; on a worldwide, perpetual, irrevocable, royalty-free basis to BMJ Publishing Group Ltd ("BMJ") its licensees and where the relevant Journal is co-owned by BMJ to the co-owners of the Journal, to publish the Work in this journal and any other BMJ products and to exploit all rights, as set out in our licence.

The Submitting Author accepts and understands that any supply made under these terms is made by BMJ to the Submitting Author unless you are acting as an employee on behalf of your employer or a postgraduate student of an affiliated institution which is paying any applicable article publishing charge ("APC") for Open Access articles. Where the Submitting Author wishes to make the Work available on an Open Access basis (and intends to pay the relevant APC), the terms of reuse of such Open Access shall be governed by a Creative Commons licence – details of these licences and which Creative Commons licence will apply to this Work are set out in our licence referred to above.

Other than as permitted in any relevant BMJ Author's Self Archiving Policies, I confirm this Work has not been accepted for publication elsewhere, is not being considered for publication elsewhere and does not duplicate material already published. I confirm all authors consent to publication of this Work and authorise the granting of this licence.

For peer review only

Title: The Potential Impacts of General Practitioners Working in or Alongside Emergency Departments in England: initial qualitative findings from a national mixed-methods evaluation

Arabella Scantlebury[1], Heather Brant[2], Helen Anderson[1], Heather Leggett[1], Chris Salisbury[3], Sean Cowlishaw[4], Sarah Voss[2], Jonathan Benger[5], Joy Adamson[1]

1. Department of Health Sciences, University of York, York, United Kingdom
2. Faculty of Health and Life Sciences, University of the West of England, Bristol, United Kingdom
3. School of Social and Community Medicine, University of Bristol, United Kingdom
4. Department of Psychiatry, The University of Melbourne, Australia
5. Bristol NHS Clinical Commissioning Group, Bristol, United Kingdom.

Corresponding author: Arabella Scantlebury, York Trials Unit, Department of Health Sciences, University of York, YO10 5DD. Arabella.scantlebury@york.ac.uk

Word count: 4385

For peer review only

ABSTRACT

Objectives: To explore the potential impacts of introducing General Practitioners into Emergency Departments (GPED) from the perspectives of service leaders, health professionals and patients. These 'expectations of impact' can be used to generate hypotheses that will inform future implementations and evaluations of GPED.

Design: Qualitative study consisting of 228 semi-structured interviews.

Setting: 10 acute NHS hospitals and the wider healthcare system in England. Interviews were undertaken face-to-face or via telephone. Data were analysed thematically.

Participants: 124 health professionals and 94 patients and carers. 10 service leaders representing a range of national organisations and government departments across England (e.g. NHS England and Department of Health) were also interviewed.

Results: A range of GPED models are being implemented across the NHS due to different interpretations of national policy and variation in local context. This has resulted in stakeholders and organisations interpreting the aims of GPED differently and anticipating a range of potential impacts. Participants expected GPED to affect the following areas: ED performance indicators; patient outcome and experience; service access; staffing and workforce experience; resources. Across these 'domains of influence' arguments for positive, negative, and no effect of GPED were proposed.

Conclusions: Evaluating whether GPED has been successful will be challenging. However, despite uncertainty surrounding the direction of effect, there was agreement across all stakeholder groups on the areas that GPED would influence. As a result, we propose 8 domains of influence that will inform our subsequent mixed-methods evaluation of GPED.

Trial registration: ISRCTN51780222.

Key words: Primary Care; Accident & Emergency Medicine; qualitative research; health policy.

For peer review only - http://bmjopen.bmj.com/site/about/guidelines.xhtml

ARTICLE SUMMARY

Strengths and limitations of the study

- A unique primary study of ten NHS case sites explores the anticipated effects of introducing General Practitioners in Emergency Departments.
- Our analysis uses a large qualitative data set and incorporates the views of multiple stakeholders.
- Data is from England only and so may not be generalizable to other healthcare settings.
- Data represents the views of those individuals who agreed to take part, and so may not be exhaustive.

BACKGROUND

Urgent and emergency care is experiencing increasing demand globally.[1] In 2019, attendances at emergency departments (EDs) in England stood at record levels. 2018-19 saw an increase of 4.4% compared with 2017-18, and 21% since 2009-2010.[2] High levels of ED occupancy lead to crowding,[3] and this can undermine patient safety, clinical outcomes and quality of care,[3-5] delay service delivery,[6] increase associated mortality and reduce patient and clinician satisfaction [7].

Numerous initiatives have been introduced to address the challenge of rising demand in ED attendance globally. [8-12] Examples of UK initiatives include the introduction of telephone advice and guidance (NHS 111/NHS Direct) and the provision of alternative facilities (e.g. walk-in centres, urgent treatment centres) for patients to access primary care for non-urgent conditions.[1, 13]

It is estimated that between 15% and 40% of patients attending the ED could be treated in general practice.[14-16] Over the past decade, EDs across the UK and Europe have started to introduce general practice (GP) services in or alongside emergency departments. [17] In addition to being introduced to try and tackle a rise in demand from perceived general practice patients, it was anticipated that introducing GPs in or alongside emergency departments would, by providing specific general practice skills and expertise, lead to improvements in patient care and control costs by reducing admission and investigation rates.[18]

For peer review only - http://bmjopen.bmj.com/site/about/guidelines.xhtml

In 2015, a review of NHS Urgent and Emergency Care in England proposed that selected patients should be directed to an alternative healthcare provider who could better meet their needs, thereby reducing ED attendances.[19] In 2017 this recommendation was translated into policy in the 'Next Steps on the NHS Five Year Forward View' stating that, "Every hospital must have comprehensive front door streaming by October 2017" (p. 15).[20] To provide financial support for the introduction of GPs working in or alongside the ED, the UK government also announced a capital fund of £100million to which hospitals in England could apply.[21-24]

Despite the recent political and financial commitment by the UK government to introducing GPs in or alongside EDs, recent guidance from the National Institute of Health and Care Excellence (NICE) stated that based on current research [25-27] there is currently 'insufficient evidence to reach a recommendation on co-located GP units.'[28] It remains uncertain how the implicit hypotheses about the effect of GPs in an ED are articulated and understood by policymakers, service leaders, health professionals and patients. These initiatives have not been subject to rigorous, independent evaluation and there is a lack of clarity regarding the assumptions and mechanism(s) through which the predicted performance benefits for these initiatives might be achieved.[29]

In this paper we report findings from qualitative data, which was collected as part of a wider mixed methods study evaluating the impact of GPs working in or alongside the ED (GPED). Further details of the GPED study are outlined in box 1 and in the study protocol.[29] This paper uses qualitative data from service leaders, health professionals and patients to explore the expected impact of introducing GPs into the ED to generate hypotheses that inform how GPED will be evaluated in subsequent research, and implemented into practice.

METHODS

Design

We completed a qualitative study consisting of interviews with service leaders, health professionals and patients from 10 case study sites (Table 1). The qualitative data reported here was collected as part of the wider GPED study (Box 1), which was approved by East Midlands – Leicester South Research Ethics Committee (ref:17/EM/0312), the University of

For peer review only - http://bmjopen.bmj.com/site/about/guidelines.xhtml

Newcastle Ethics Committee (Ref: 14348/2016) and also received HRA Approval (IRAS: 230848 and 218038).

Box 1. The GPED Study

Objectives: To evaluate the impact of GPED on patient care, the primary care and acute hospital team and the wider urgent care system.

Design: A mixed methods study consisting of three work packages.

- Work Package A: Mapping, description and classification of current models of GPED in all EDs in England, and interviews with key policymakers to examine the hypotheses that underpin GPED.
- Work Package B: Quantitative analysis of national data to measure the effectiveness, costs and consequences of the GPED models identified in work package A using retrospective analysis of Hospital Episode Statistics.
- Work Package C: detailed mixed methods case studies of different GPED models consisting of: non-participant observation of clinical care, semi-structured interviews with staff, patients and carers, workforce surveys with emergency department staff and analysis of locally available routinely collected hospital data.

PPI: A study PPI group has contributed to research design and materials and data interpretation and dissemination through a series of face-to-face workshops.

Trial status: In progress (ISRCTN51780222)

Funder: National Institute for Health Research (NIHR) Health services and Delivery (HS&DR) Programme.

For peer review only - http://bmjopen.bmj.com/site/about/guidelines.xhtml

Table 1 Data collection

	Service leaders (national)	Case studies (10 hospital sites)
Total number of participants interviewed	10	Health Professionals (124)<br>Patients/carers (94)
Interview type	Semi-structured telephone interviews	Semi-structured face-to-face and telephone interviews
Aim	In-depth understanding from key informants	In-depth understanding from selected case sites
Job roles represented	Department of Health and Social Care, NHS England, NHS Improvement, Royal College of Emergency Medicine	GPs working in the ED, ED doctors (juniors, registrars, consultants), Nurses (streaming, triage, minor injuries, Emergency Nurse Practitioners), ED managerial and clinical leads, clinical directors

Sampling and recruitment

Data were collected from 10 case study sites. Sites were selected purposively to ensure maximum variation according to: GPED model; GPED duration; geographical location; deprivation index and ED volume (ED attendances).[30] Participants were sampled opportunistically by the research team, whilst undertaking on-site data collection. Service leaders were contacted directly via email.

Data collection

Telephone interviews with service leaders were conducted between December 2017 and January 2018 following informed verbal consent. During interviews participants were asked to describe: their involvement in GPED and background to the policy as well as the expected impact of GPED and any potential unintended consequences (Supplementary material 1).

Case study interviews with patients and health professionals were largely conducted face-to-face at hospital sites during GPED study data collection. Some interviews were conducted

For peer review only

via telephone at the request of the participant. Written informed consent was provided by all participants and all interviews were audio-recorded. Data collection took place between October 2017 and November 2018 at 10 EDs throughout England. Interviews with health professionals, patients and carers were semi-structured and followed a topic guide (supplementary material 2-7). During interviews health professionals were asked: their current role in ED; details of their GPED model; expected impact. Patients and carers were asked to describe why they chose to attend the ED as well as their experiences. Patients were also asked about their views on introducing GPED and its potential impact.

Analysis

AS, HA, HL and members of the wider GPED research team undertook data collection and analysis. HA is a registered nurse with experience of working in primary care. All other members of the research team involved in data collection and analysis are health services researchers.

Analysis was facilitated by use of the qualitative data management programme NVIVO. After familiarisation, a coding framework was developed through a series of roundtable discussions by the research team and was continually refined and revisited during researcher meetings on an on-going basis throughout data collection and analysis. This framework was used to produce a series of summaries and pen portraits to describe each case site,[21] which informed a final thematic analysis during which themes were refined further for the purpose of this paper.[22] All participants and case sites were allocated unique personal IDs, to protect anonymity and confidentiality. Unless otherwise specified we use the term staff to collectively refer to GP and ED staff throughout the results section.

Patient and Public Involvement

Ten public contributors with experience of using ED services have been directly involved in the design, development and interpretation of the GPED study. In addition to attending external steering group meetings and supporting the development of our original application for research funding and key study materials (e.g. information sheets), our ten public contributors have participated in regular workshops throughout the GPED study. During these workshops, public contributors were given copies of anonymised interview transcripts along with pen portraits from two of our study sites. Public contributors initially

For peer review only

For peer review only - http://bmjopen.bmj.com/site/about/guidelines.xhtml

discussed how they interpreted the data, before being asked to consider whether their own interpretations resonated with the research team's framework. Additional workshops are also being held to discuss the wider GPED study's findings where both quantitative and qualitative data will be presented and discussed with the group.

RESULTS

Service leaders and site staff perceived the national implementation of GPED as a response to increasing pressure on EDs, with a lack of supporting research evidence. Many viewed GPED as a top-down, generalised strategy that had been imposed on them without consideration of local context. Ultimately, variations in local context, ED demand and existing GP services in or alongside the ED meant it was not considered possible to implement the same system everywhere. This resulted in a "proliferation of different models", which in turn implied that the impact of GPED on ED performance would vary substantially.

Our qualitative data highlight the challenges associated with a top-down national policy that is implemented in different ways according to local context. We hope to demonstrate the complexity and uncertainty this brings when trying to predict and then evaluate how the policy may impact patients, Emergency Departments and the wider urgent care system. Our results are therefore presented as a series of areas that stakeholders believed would be affected by the introduction of GPED, and the direction of the anticipated effect.

Performance indicators

The premise that ED staff and GPs have inherently different approaches to risk was central to the concept of GPED. GPs were perceived to frame health and illness in a different way to ED staff, with the 'wait and see' culture of primary care leading many to view GPs as more 'risk tolerant' and more appropriately qualified to care for lower acuity patients than their 'risk averse' ED colleagues. This in turn was thought to be beneficial for GPED by making GPs less likely to order unnecessary investigations, or admit or refer lower acuity patients unnecessarily, thereby reducing the time spent in the ED and enhancing patient flow. Despite this general articulation of potential performance benefits, there was significant uncertainty about the impact of GPED within the local systems included in our case studies. One of the main areas of disagreement among site staff and service leaders

For peer review only - http://bmjopen.bmj.com/site/about/guidelines.xhtml

was whether GPs were more tolerant of risk and if so whether this would have adverse consequences for patient safety. This resulted in variation in GPED models across sites. Individual views largely varied according to the degree of integration and the specific role of GPs within the system – making it difficult to identify generalised predictions relating to the potential impact of GPED.

Use of investigations

Many participants were accepting of models that asked GPs to work in a hybrid ED-GP role and encouraged GPs to ‘go native’, becoming highly integrated within ED teams. Some models were based on the premise that GP access to investigations was crucial to GPED effectiveness – with concerns that the potential scope of GPED would be limited by GPs not being able to undertake investigations and refer to specialties. In contrast, other GPED models limited GPs to working as they would in the community, and service leaders felt strongly that for the model to run effectively GPs and the ED should work separately. There was an idea that GPs ‘going native’ would encourage them to behave in a similar way to ED doctors, thereby negating any assumed benefits from GPs’ different attitudes to risk, investigation and referral. Therefore, prior expectations relating to unnecessary testing were mostly factored into the GPED model at the outset.

Hospital admissions and the 4-hour target

Reducing hospital admissions and improving performance against the ‘four-hour standard’ (that 95% of ED patients should be discharged, admitted or transferred within 4 hours of arrival) were often quoted as among the potential benefits of GPED. However, this was not universally accepted. For example, some felt that admissions would not be affected, because the population being targeted are not those that would normally be admitted from the ED. Equally, targeting primary care patients was welcomed by ED managers, as although GP patients can be dealt with quickly in theory, in many localities these patients are present in high volumes and were perceived to be at risk of breaching the four-hour standard. However, some feared there might be an unintended worsening effect – diverting people with minor conditions that are theoretically quick to resolve increases the acuity of the remaining ED patient workload. If the ED is left with only high acuity patients, there is a

For peer review only

For peer review only - http://bmjopen.bmj.com/site/about/guidelines.xhtml

possibility that both the time spent in the ED and the proportion of patients who are admitted will increase, worsening the reported “four hour” performance.

When stakeholders discussed possible effects of GPED on performance indicators it was not always clear, and was not model dependent, whether GPED streamed patients were to be included or excluded from the ED figures, and assumptions regarding this influenced participants’ views. Generally, performance indicators were considered blunt tools with which to evaluate impact, reflecting potential measurement issues and artefacts rather than good clinical practice. It was also anticipated that the ‘visibility’ and impact of GPED would be obscured by a year-on-year increase in patient attendances and hospital admissions (table 2).

Table 2 Arguments proposed for the potential impact of GPED on ED performance

ED performance and performance indicators				
Potential impact	Positive	Negative	No difference	Exemplar quote
Use of investigations/ testing	Risk tolerant nature of GPs makes them suitable for working alongside the ED – less likely to order investigations unnecessarily	GPs lack skills to work in ED<br><br>By ‘going native’ and having access to investigations/testing GPs may lose their unique skills and work similarly to ED doctors	Whether GPs were given access to investigations varied depending on the GPED model in place and so any impacts associated with this would be negligible.	*“It was suggested that those problems could be better dealt with by primary care clinicians who had the appropriate skills for the job and would be perhaps confident about seeing and treating and discharging without over-investigation.” (Rowan. Staff interview, 07)*
Admissions	Avoid unnecessary admissions of lower acuity patients and improve patient flow	If the ED is left with only high acuity patients the proportion of ED attendances who are admitted will increase	Admissions not affected as the population targeted is not those that would be admitted from ED.	*“But I can’t pretend that I think it will make a massive difference on admissions, because the people who are waiting for admission are*

For peer review only

For peer review only - http://bmjopen.bmj.com/site/about/guidelines.xhtml

				very largely a different group of people you see." (Service Leader interview, 02)
Waiting time/ 4 hour KPI	Streaming primary care patients to GP (the most appropriate clinician), reduces the risk of breaching the four hour target as lower acuity patients are high in volume and occupy a lot of clinician time	Diverting patients with minor conditions who are theoretically quick to resolve will increase the acuity of ED work and make improvements in the "4 hour target" less likely. Higher acuity patients are considered more complex and so take longer to manage, increasing the potential for breaching the target	Number of minor breaches that would need to be converted is too large to see any improvement in "4 hour performance"	*"In theory, if you've taken all the minors, all the sort of streamed patients and minor cases out, you'll have ... your staff that are there will be able to devote more time dealing with the majors. And similarly they were hoping that you'd be reducing the volume of patients coming through there but you would hopefully be able to increase the rate the patients were seen. So you would reduce the number of breach patients coming through the main ED department." (Service Leader interview, 07)*

Patient outcome and experience

A process of front door "streaming" of patients on arrival at the ED was intended to facilitate the identification of low acuity patients and match them with the availability and skills of the treating clinician (e.g. a general practitioner). This differs from 'triage', which although often used interchangeably with streaming, refers to the identification of high

For peer review only - http://bmjopen.bmj.com/site/about/guidelines.xhtml

acuity patients to ensure that more urgent cases are identified and treated in a timely way. By introducing front door streaming, [31] EDs were expected to see improvements in patient outcomes (some of which are reflected in the performance standards) and experience (table 3). Streaming lower acuity patients to a GP was anticipated to improve patient care by enabling ED staff to focus on higher acuity patients and ensure that GP acuity patients are treated in GPED rather than being 'sent round the houses'. Patients were aware of the significant resourcing and financial pressures placed on the NHS and so saw value in placing GPs in the ED.

There were concerns, however from service leaders and ED staff, that patient flow could be negatively affected by GPED with a backlog created by patients being required to disclose clinical information on multiple occasions before seeing a GP, or that GPED patients would prevent those with higher acuity needs being seen in a timely manner due to beliefs that GPED may increase the number of patients attending ED and associated crowding (see below).

There was strong and divided opinion between staff groups and even service leaders as to what is considered a 'GPED appropriate' patient. These opinions were often underpinned by cultural differences between GPs and ED staff and staff perceptions regarding professional competencies, boundaries and skillsets. ED staff in particular made certain assumptions about the skill set of GPs, which influenced these views. In some cases, GPs were perceived to lack the appropriate skills and experience to work in the ED, which in turn was felt to limit the potential effectiveness of GPED. Models that required GPs to 'go native' were thought to ask GPs to work beyond their clinical competency, with some staff claiming that GPs are not up to date with ED knowledge, and lacking in key clinical skills such as x-ray interpretation and suturing. There were also concerns that GPs may not recognise higher acuity patients, with associated risks to patient safety.

Table 3 Arguments proposed for the potential impact of GPED on patient outcome/experience

Patient outcome and Experience				
Potential impact	Positive	Negative	No difference	Exemplar quote
Streaming patients to the appropriate clinician	Improved flow of patients through the system	Backlog created by patients having to disclose information on multiple	Annual growth of ED workload may mask impact	*"Intended impact was to divert as many patients who were able to be streamed to a primary care service, away from the A&E and ED*

For peer review only - http://bmjopen.bmj.com/site/about/guidelines.xhtml

		occasions before seeing GP.	of GPED on performance	*departments, reducing then, surge of patients through and ensuring that patients could be seen quickly and effectively both in A&E and ED, but also in the located primary care services."(Service Leader interview, 10)*
Patient experience	Improved patient experience by streaming patients to a GP since this avoids them being 'sent around the houses' and/or waiting in lengthy ED queues, enabling quicker assessment and discharge.	GPED patients may prevent those with higher acuity being seen in a timely manner – GPED may increase the number of patients attending ED		*"I'd like to think if it was working out as we'd originally envisaged that trusts would be able to flow people through the main ED departments much quicker. So we would see reduced breaches. So the four-hour performance would improve but similarly patient experience would significantly improve because you would hopefully be reducing the number of delays to patients getting treated. So hopefully it would just be freeing up the ED department, by taking the streamed patients out. So that's what I was hoping we would see.' (Service Leader interview 07)*
Value of GP	Patients saw value in GPED due to resourcing and financial pressures on NHS	GPs lack appropriate skills and experience to work in ED.		*"What's nice is it takes the pressure off the, er, general A&E and actually emergencies can get deal with emergencies and not get clogged up." (Teak. Patient interview, 021)*

For peer review only

Service Access

There was divided opinion as to how GPED may affect ED attendance (table 4). Despite one of the aims of GPED being to create a more efficient service, both staff and patients were concerned that GPED may become a product of its own success by encouraging people to attend ED with primary care problems repeatedly, and that GPED would become a replacement GP service. It was felt that despite any 'educational' component, whereby patients are encouraged to use their own GP when attending GPED, the fact that GPED guaranteed same day access to a GP was in conflict with this message, and could encourage 'inappropriate' attendance with routine rather than urgent care needs. Concerns that GPED

For peer review only - http://bmjopen.bmj.com/site/about/guidelines.xhtml

could create additional demand on the ED were supported by anecdotal reports from established GPED models highlighting that the volume of patients had increased since introduction. This rise was attributed to the service generating new demand from primary care patients. Others highlighted the potential influence of general practice opening times; because primary care patients tend to present out of hours, GPED could cause peaks in ED attendance when general practice surgeries are closed.

Yet this view was not universal, service leaders provided various reasons why the policy was unlikely to cause an increase in ED attendance. For example, service leaders argued that given the average person attends the ED less than once a year, it is unlikely that they would start using ED as their main access to general practice. Additionally, as many ED patients present with higher acuity, GPED was not expected to be a supply driver in the same way as a walk-in centre. To this end, GPED was not viewed as being about access to GPs, but about streaming patients to the most clinically appropriate professional. A lack of advertising, the fact that most cases would still be treated in the ED and a lack of patient awareness of GPED was also perceived to mean that GPED would have a negligible impact on demand.

Table 4 Arguments proposed for the potential impact of GPED on ED attendance

Service Access				
Potential impact	Increase	Decrease	No difference	Exemplar quote
GPED as a replacement primary care service	GPED becomes a replacement GP service		Streaming patients to most appropriate professional<br><br>Average person uses ED less than once a year so unlikely to become the main source of general practice	*"I guess my personal view is I think they're probably putting GPs on hospitals because they've realised people are fed up of waiting to get an appointment at the GPs and they're going to hospitals, so they're not really fixing the problem there." (Redwood. Patient interview, 02)*
Increase 'inappropriate' attendance	Same day access to a GP may encourage 'inappropriate' attendance	Many patients present with high acuity needs, so not the same as a		*"But I think, I think what it, what it does do is that, it further reinforces the concept if*

For peer review only - http://bmjopen.bmj.com/site/about/guidelines.xhtml

		walk-in centre in terms of supply.		*you've got an urgent and emergency care problem you go to ED, because not only is the ED and x-rays and prescriptions there and all the rest of it there, but now you've got primary care there as well….I kind of think it acts as a supply site driver."* *(Service Leader interview, 005)*
Increase demand on ED	Peaks in attendance when general practice surgeries are closed.		Patients unaware of GPED service	*"It hasn't been well publicised … patients, I don't think most patients will be aware of it. I think that given they get treated in an emergency department they will probably not recognise that there is, that there's a GP service …" (Service Leader interview, 01)*

Staffing and workforce experience

Staffing issues dominated discussions about the potential impact of GPED, and were seen to pose a major threat to its success (table 5). Services leaders and site staff expressed concern that GPED could draw GPs away from primary care and cause competition for GP staff. Consequently, GPED was perceived to have the potential to worsen general practice staffing issues, which in turn could increase waits for a GP appointment and further encourage people to attend ED.

GPED was considered an attractive prospect for those GPs seeking portfolio careers and wishing to expand their practice, knowledge and skills. Traditional general practice was seen as a more stressful and less attractive workplace than newer service models. This was due to several pressures including increasing volume and complexity of workload and depleted

For peer review only - http://bmjopen.bmj.com/site/about/guidelines.xhtml

community and social care provision. There was some debate as to how the flexible hours associated with GPED would impact on job satisfaction. For example, some anticipated that this flexibility would make it easier to fill rotas, whilst others felt that shift working goes against one of the main reasons why people choose to be a GP.

Table 5 Arguments proposed for the potential impact of GPED on staffing and experience

Staffing and workforce experience			
Potential impact	Positive	Negative	Exemplar quote(s)
GPs want to work 'beyond the walls of the surgery'	GPED is an attractive place to work for those wanting portfolio careers.	Working 'beyond the walls of the surgery' is not appealing to all and may cause competition for GP staff between primary and secondary care	*"A concern [is] that it would, it would spread the primary care resource more thinly, so it would be less able to respond to, you know, would be less able to respond to sagittal primary care demand..."(Service Leader interview, 05)*
Flexible working hours	Flexible working hours may make it easier to fill rotas	Working out of hours is a deterrent for those who chose to work in general practice	*"Just because I'm a locum I can avoid doing nights, and chose not to do nights." (Chestnut. Staff interview, 22)*
Locum working	Working on a locum or ad hoc basis can be attractive to some and may mitigate against GP staffing issues.	Difficult to ensure the quality of locum staff and inconsistent workforce supply negatively affects collaborative working between ED and GPs	*"The barriers, yes. Often, the GPs are not there all the time, it's not the same person. They're often locum. So, the GP will, sort of, arrive, go straight into their room and then stay in the room unless you call them out for huddle ….. whereas A&E nurses and all of our doctors are all quite social, we're a team, we're really visible to each other. I think just the mentality of a GP is you sit in your room all day, don't you, on your own?" (Nutmeg. Staff interview, 15)*

Many staff perceived GPED to have training and educational benefits for junior doctors who would, in some models, become more confident about discharging patients and build up their primary care knowledge (table 6). Conversely, diverting patients with minor conditions to GPED was seen to have benefits for ED juniors and trainees by exposing them to more acutely ill patients.

However, there was a perceived lack of suitably qualified GPs with the necessary skills and experience to work effectively in GPED. Site staff placed importance on making GPED an

For peer review only - http://bmjopen.bmj.com/site/about/guidelines.xhtml

attractive place to work and ensuring that GPs feel valued, supported and appropriately remunerated for effective implementation. Emphasis was also placed on ensuring GPs feel protected and supported to work within their scope of practice. As a result, some felt that GPs needed to be upskilled or would require extra training. To compensate for this, some respondents emphasised the importance of recruiting experienced GPs, who had previously worked in the ED, or employing GPs that were trained at their hospital site as juniors.

There was also concern that experienced nursing staff may prefer to work in GPED due to 'better' working hours and it being perceived as an easier job. This not only has implications on ED staffing, but on streaming, which many felt should be undertaken by an experienced nurse. However, some nurses perceived streaming to be a waste of their clinical skills and believed that it took them away from their central role and left ED short-staffed. ED nurse practitioners were also concerned that although they continued to see patients with minor injuries, minor illnesses would be streamed to GPED, which could result in de-skilling of the ED nursing workforce.

Table 6 Arguments proposed for the potential impact embedding GPs in ED teams

	Integrating GPs as part of the ED team		
Potential impact	Positive	Negative	Exemplar quote
Training and clinical skills	Benefits for improving team working and skill mix. Training and educational benefits for junior doctors and GPs.	GPs may lack appropriate skills/experience to work in ED	*"Yes, knowledge and experience. GPs could teach about headaches to the primary care nurse and us, if we wanted to help out a little bit, to bring on new nurses who are coming through and learn. Then you could develop majors practitioners, bring them through. Do teaching and education, bring minors and- it would be a perfect bed of opportunity." (Rowan. Staff interview, 20)*
Deskilling of GP and nursing workforce	Nurses prefer to work in GPED	Integrating GPs may cause deskilling.<br><br>Negative views on streaming and the potential for GPED to	*"There's a risk that the GPs who are then working on a consistent basis within an emergency department or as*

For peer review only

For peer review only - http://bmjopen.bmj.com/site/about/guidelines.xhtml

		deskill the nursing workforce by diverting minor illnesses to GPED.	*part of.... that they can go native within that setting and actually take on more of the, qualities that you might expect to see, in other emergency department staff and actually lose the characteristics that you might expect to see of a GP."(Service Leader interview, 10)*

Resources

Staff and patients predicted that GPED would incur higher costs due to the cost of GP employment, and placed importance on ensuring staffing and resources are carefully matched (table 7). Staff considered GPs a costly resource and felt that GPs needed to demonstrate their effectiveness. Furthermore, the employment of locums and agency staff to fill these positions was expected to lead to greater costs. There were some concerns that the funding could be better spent improving general practice provision, which may lead to the same outcome. Incidental costs such as paying for training and the set-up and management of new IT systems was considered an added cost and time burden that staff felt had not always been taken into consideration.

Positively, GPED was seen by some as a cost-effective initiative through its presumed effect of reducing hospital admissions and unnecessary patient investigations. If patients were seen by a GP this would release ED staff to treat more unwell patients with a potential cost saving arising from the more effective use of staff resources (i.e. patients being seen by the most appropriate staff member).

Table 7 Arguments proposed for the potential impact of GPED on resources

	Resources		
Potential impact	Positive	Negative	Exemplar quote
Costs	Reduction in hospital admissions and patient investigations.<br>Streaming patients to the appropriate clinician may	GPs are a costly resource.<br>Reliance on locums and agency staff.	*"Costs had a massive factor in it. Staffing, we kind of have to work around the cost. So sometimes*

For peer review only

For peer review only - http://bmjopen.bmj.com/site/about/guidelines.xhtml

	result in cost-savings through more effective use of staff resources.		*it's, painfully, not for how many you should have to be able to run the department, it's how many can we afford to have to run the department safely."* (Chestnut. Staff interview. 023)
Infrastructure		Training and IT set-up and management.	*"The training was, I have to say, on the computer system, not great. I tried to get some IT training on the system. The IT department said there wasn't any training available, but they'd let me know when there was."* (Redwood, Staff interview.007)

DISCUSSION

Main findings

Since the 2017 implementation of "comprehensive front door streaming", supported by capital funding [14-18], a variety of different GPED models have been introduced throughout the NHS. This is in part a response to varying local needs and contexts, and also different interpretations of what GPED means on a practical level. This has resulted in disagreement at an individual, stakeholder and organisational level about the purpose and anticipated benefits and dis-benefits of GPED and a lack of clarity about the impact of introducing GPED on these effects. Indeed, for each domain of influence we present there were, in most cases, arguments for positive, negative and no effects of GPED (tables 2-6).

Despite disagreeing about the 'direction of effect,' stakeholders agreed about which areas of the healthcare system and patient care were most likely to be impacted by GPED. This has enabled us to generate 'domains of influence', which will form the basis of our subsequent mixed methods evaluation of the impact of GPED on patient care, the general

For peer review only - http://bmjopen.bmj.com/site/about/guidelines.xhtml

practice and acute hospital team and the wider urgent care system during the wider GPED study (Box 2).

Box 2: GPED Domains of influence

- Performance against the four hour target/waiting time
- Use of investigations
- Hospital admission
- Patient outcome/experience
- Service access
- Staffing
- Workforce
- Resource use/cost

Whilst the domains of influence provide the foundation for our wider mixed methods evaluation of GPED, a lack of agreement surrounding the policy's aims, coupled with uncertainty as to how the anticipated impacts will be achieved, poses a significant challenge when evaluating whether GPED can be considered a successful national policy.

It is also unclear whether the success of GPED should be determined by its effect on EDs or the wider healthcare system. This warrants careful consideration since some domains, such as ED costs or performance, may be improved at the expense of the wider NHS. Additionally, many of the differences in opinion surrounding the potential impact of GPED are underpinned by confusion as to whether patients attending the GPED are considered part of, or separate from the denominator used for measuring ED performance. This has implications for understanding the effect of GPED on key performance indicators, particularly the "4 hour target".

Comparison with existing literature

In 2010 Carson et al explored rationales for the introduction of GPED through an online survey. They report that "The main reason was to meet the needs of patients or improve quality of care. This was followed by achieving the four-hour target and reducing cost."[18]

Similar assumptions have persisted, and were seen to be drivers of the policy initiative to roll out GPED in all EDs across England. Benefits of GPED, particularly to address the increasing demand in emergency care, were perpetuated through rhetoric presented in the

For peer review only - http://bmjopen.bmj.com/site/about/guidelines.xhtml

national press [32], clinical press releases [33], medical journals [23, 34] and within the policy documents produced at the time [35, 36].

Early studies appeared to underpin some of these assumptions. Evaluations of early adopters in the UK and Europe suggested that GPs in the ED could “result in reduced rates of investigations, prescriptions, and referrals”,[9, 37] increase patient satisfaction,[8] and offer patients a greater range of healthcare provision.[38] However, these studies have generally been of poor quality.

More recently, these assumed benefits have been challenged. A realist review concluded that despite a reduction in process time for non-urgent patients this does not necessarily increase capacity to care for the sickest patients.[31] The main cause of ED crowding is a lack of beds and congestion in the flow of sicker patients rather than absolute attendance numbers.[39] In addition, GPED may encourage patients to present to the ED with a primary care problem, with consequent increases in ED attendance.[26, 40]

To date, reviews that examine GPED in more detail have concluded that there is insufficient evidence to support national policy or local system change.[25, 26, 41] Two Cochrane reviews (2012 and 2018) concluded that there was “insufficient evidence upon which to draw conclusions for practice or policy regarding the effectiveness and safety of care provided to non-urgent patients by GPs versus EPs in the ED to mitigate problems of overcrowding, wait-times and patient flow” (p.2).[27, 42]

Strengths and Limitations

The ‘domains of influence’ that we have identified in this paper were generated from a large evaluation that used ‘big qualitative data’ (228 interviews) and the views of multiple stakeholders. This provided a rich and nuanced understanding of the complexity surrounding a current national policy – GPED. Our data apply to England only, and so may not be generalizable to other healthcare settings. In addition, we could only interview those who agreed to take part, and whilst we did not ‘strive for saturation’, the range of views may not exhaustive. However, our maximum variation approach did achieve data that spans a very wide range of individuals. [30] The detail we have obtained has enabled us to propose the domains of influence that will be used to inform our wider GPED study, the aim of which is to evaluate the impact of GPED on each of the domains of influence in detail. It could be

For peer review only - http://bmjopen.bmj.com/site/about/guidelines.xhtml

argued that the data we present here represents the inherent uncertainty and resistance to change that most healthcare policy encounters prior to or during early implementation, and so is representative of typical ‘teething problems.’ However, while it is assumed that such issues will improve over time, recent research suggests that issues that are identified early in the implementation process often persist long after establishment.[43] It is our hope that by identifying ‘domains of influence’, rather than a set of hypotheses, we have mitigated against this and have identified many of the key areas that the GPED policy is likely to affect, whilst providing a framework to guide our forthcoming mixed methods evaluation.

CONCLUSION

In 2017, a significant financial commitment to support hospitals introduce GPs in ED was made in a direct attempt to address growing concerns surrounding the pressures on emergency departments. However, the reality of introducing GPs in ED is complex. Throughout the NHS, the policy is being interpreted differently, which has created a range of GPED models to be implemented into ever-changing and variable local contexts. This variation both in terms of how the policy is being interpreted and introduced, different ‘baseline levels’ of GPED and the lack of agreement from stakeholders surrounding the potential benefits and dis-benefits of the policy, mean that the impact of GPED is difficult to predict. However, our findings suggest that GPED will affect 8 key areas. These ‘domains of influence’ will be used as the foundation for our subsequent mixed methods evaluation.

ACKNOWLEDGEMENTS

The authors would like to thank the participants for their involvement in the study, the GPED public contributors and wider GPED research team.

FUNDING STATEMENT

This work was supported by the National Institute for Health Research (NIHR) Health Services & Delivery Research (HS&DR) Programme, project number 15/145/06.

DISCLAIMER

The views and opinions expressed therein are those of the authors and do not necessarily reflect those of the HS&DR Research Programme, NIHR, NHS or the Department of Health.

For peer review only

For peer review only - http://bmjopen.bmj.com/site/about/guidelines.xhtml

COMPETING INTERESTS

Jonathan Benger is seconded part-time to the post of interim Chief Medical Officer at NHS Digital. All other authors declare no conflict of interest.

ETHICS APPROVAL

Approval for the study has been obtained from the Health Research Authority (HRA) (IRAS: 230848 and 218038). The protocol was reviewed and received a favourable opinion from the NHS East Midlands – Leicester South Research Ethics Committee REC: 17/EM/0312 and the University of Newcastle Ethics Committee (Ref: 14348/2016) a

PROVENANCE AND PEER REVIEW

Not commissioned; peer reviewed for ethics and funding approval prior to submission.

DATA SHARING STATEMENT

The data set which we have acquired will not be available as our ethical approval does not permit the sharing of the entire dataset.

AUTHOR CONTRIBUTIONS

AS drafted the manuscript, undertook data collection and analysis. HA and HL, undertook data collection and analysis and critically appraised the manuscript. HB, SC, CS and SV critically appraised the manuscript. JA and JB helped to draft the manuscript – JA also undertook analysis. JA, JB, SC, CS and SV designed the study. All authors have reviewed and approved the final manuscript.

REFERENCES

1. Berchet, C., *Emergency Care Services: Trends, Drivers and Interventions to Manage the Deman*. 2015, Organisation for Economic Co-operation and Development: Paris, France.
2. NHS Digital, *Hospital Accident & Emergency Activity 2018-19*. 2019, NHS Digital: England.
3. Bernstein, S.L., et al., *The Effect of Emergency Department Crowding on Clinically Oriented Outcomes.* Academic Emergency Medicine, 2009. **16**(1): p. 1-10.
4. Collis, J., *Adverse effects of overcrowding on patient experience and care.* Emergency nurse, 2010. **18**(8).
5. Morley, C., et al., *Emergency department crowding: A systematic review of causes, consequences and solutions.* PloS one, 2018. **13**(8): p. e0203316-e0203316.
6. Rasouli, H.R., et al., *Outcomes of Crowding in Emergency Departments; a Systematic Review.* Archives of academic emergency medicine, 2019. **7**(1): p. e52-e52.
7. Higginson, I., *Emergency department crowding.* Emergency Medicine Journal, 2012. **29**(6): p. 437-443.

For peer review only - http://bmjopen.bmj.com/site/about/guidelines.xhtml

504 8. Boeke, A.J.P., et al., *Effectiveness of GPs in accident and emergency departments.* British Journal of General Practice, 2010. **60**(579): p. e378-e384.
506 9. Wang, M., et al., *Hospital-integrated general practice: a promising way to manage walk-in patients in emergency departments.* Journal of Evaluation in Clinical Practice, 2014. **20**(1): p. 20-26.
509 10. Ablard, S., et al., *What does the ideal urgent and emergency care system look like? A qualitative study of service user perspectives.* Emerg Med J, 2020. **37**(4): p. 200-205.
511 11. Uthman, O.A., et al., *General practitioners providing non-urgent care in emergency department: a natural experiment.* BMJ Open, 2018. **8**(5): p. e019736.
513 12. Jones, M., *Walk-in primary medical care centres: lessons from Canada.* BMJ, 2000. **321**(7266): p. 928-931.
515 13. Salisbury, C., et al., *The impact of co-located NHS walk-in centres on emergency departments.* Emergency medicine journal : EMJ, 2007. **24**(4): p. 265-269.
517 14. NHS England, *Transforming urgent and emergency care services in England. Urgent and emergency care review end of phase 1 report*, in *High quality care for all, now and for future generations*. 2013, NHS England: London.
520 15. Murphy, K. and C. Mann, *Time to Act–Urgent Care and A&E: the patient perspective*. 2015, Royal College of Emergency Medicine & The Patients Association: London.
522 16. Thompson, M.I.W., et al., *Suitability of emergency department attenders to be assessed in primary care: survey of general practitioner agreement in a random sample of triage records analysed in a service evaluation project.* BMJ Open, 2013. **3**(12): p. e003612.
525 17. Yarmohammadian, M.H., et al., *Overcrowding in emergency departments: A review of strategies to decrease future challenges.* Journal of research in medical sciences : the official journal of Isfahan University of Medical Sciences, 2017. **22**: p. 23-23.
528 18. Carson, D., H. Clay, and R. Stern, *Primary care and emergency departments: report from the Primary Care Foundation. Mar 2010*. 2010, Primary Care Foundation: London.
530 19. Urgent and Emergency Care Review Team and Emergency Care Intensive Support Team, *Transforming urgent and emergency care services in England. Safer, faster, better: good practice in delivering urgent and emergency care. A guide for local health and social care communities*. 2015, NHS England: London.
534 20. NHS England, *Next Steps on the NHS Five Year Forward View*. 2017: England.
535 21. Department of Health and Social Care, *Funds for next winter will ease pressure on accident and emergency (A&E) departments in England.* 2017: London.
537 22. HM Treasury, *Spring Budget 2017*. 2017: London.
538 23. Iacobucci, G., *All emergency departments must have GP led triage by October.* BMJ, 2017. **356**: p. j1270.
540 24. Iacobucci, G., *NHS gets £100m budget "sticking plaster" to help manage emergency demand.* BMJ, 2017. **356**: p. j1248.
542 25. Turner, J., et al., *What evidence is there on the effectiveness of different models of delivering urgent care? A rapid review*. Health Services and Delivery Research. 2015: NIHR Journals Library, Southampton (UK).
545 26. Ramlakhan, S., et al., *Primary care services located with EDs: a review of effectiveness.* Emergency Medicine Journal, 2016. **33**(7): p. 495-503.
547 27. Gonçalves-Bradley, D., et al., *Primary care professionals providing non-urgent care in hospital emergency departments.* Cochrane Database of Systematic Reviews, 2018(2).
549 28. National Institute for Health and Care Excellence, *GPs within or on the same site as emergency departments*. 2017.
551 29. Morton, K., et al., *General practitioners and emergency departments (GPED)—efficient models of care: a mixed-methods study protocol.* BMJ Open, 2018. **8**(10): p. e024012.

For peer review only - http://bmjopen.bmj.com/site/about/guidelines.xhtml

30. Braun, V. and V. Clarke, *To saturate or not to saturate? Questioning data saturation as a useful concept for thematic analysis and sample-size rationales.* Qualitative Research in Sport, Exercise and Health, 2019: p. 1-16.
31. Cooper, A., et al., *The impact of general practitioners working in or alongside emergency departments: a rapid realist review.* BMJ Open, 2019. **9**(4): p. e024501.
32. Campbell, D., *NHS chiefs order hospitals to begin urgent overhaul of A&E care*, in *The Guardian*. 2017, The Guardian: London, UK.
33. Royal College of Emergency Medicine and Urgent Health UK, *The Royal College of Emergency Medicine and Urgent Health UK call for the colocation of urgent care services with A&E departments* 2015: RCEM.
34. Wickware, C., *Nearly 420 extra GPs needed across country to deliver A&E streaming service* in *Pulse*. 2017.
35. Stevens, S.a.M., J, *Action to get A&E performance back on track*, A.N.P.T.C. Executives, et al., Editors. 2017, NHS England and NHS Improvement.
36. Department of Health and Social Care., *A&E departments to get more funding: Funds for next winter will ease pressure on accident and emergency (A&E) departments in England.* 2017: GOV.UK
37. Dale, J., et al., *Primary care in the accident and emergency department: II. comparison of general practitioners and hospital doctors.* BMJ, 1995. **311**(7002): p. 427-430.
38. Bickerton, J., et al., *Streaming primary urgent care: a prospective approach.* Primary Health Care Research & Development, 2012. **13**(2): p. 142-152.
39. Oliver, D., *David Oliver: Why force GP streaming on NHS emergency departments?* BMJ, 2020. **368**: p. m992.
40. Blunt, I.E., N and Merry, L., *What's Behind the A&E 'Crisis'?* , N. Trust, Editor. 2015.
41. Fisher, J., B. Salman, and M. Cooke, *A rapid review exploring the interface between primary and emergency care in England.* Primary Care and Emergency Departments: Primary Care Foundation, 2010.
42. Khangura, J.K., et al., *Primary care professionals providing non-urgent care in hospital emergency departments.* Cochrane Database of Systematic Reviews, 2012(11).
43. Anderson, H., Y. Birks, and J. Adamson, *Exploring the relationship between nursing identity and advanced nursing practice: An ethnographic study.* Journal of Clinical Nursing, 2020. **29**(7-8): p. 1195-1208.

For peer review only

For peer review only - http://bmjopen.bmj.com/site/about/guidelines.xhtml

GPED: System Leader Interviews

What is your current role and what has your role been regarding introduction of GPs into EDs?

Tell us the background to the concept of introducing GPs into EDs as you see it
• Who have been key stakeholders in the idea
• What do they hope to achieve
• Where did it originally come from
• How it fits with other services e.g. walk in centres, 111, out of hours GP
• Have lessons learnt from the experience of introducing other services been incorporated

What is your sense of the 'buy in' from GPs?
• Sustainability (lack of GPs)
• Desirable role for GPs
• What are the challenges/benefits for GPs in this role
• Terms and conditions (e.g. employer, indemnity)

Why do you think the government have decided to invest in GPED?

Describe the different models of GPED care that you are aware of having been/going to be implemented
• Have you got a sense of which might work better (according to what outcomes)?

What do you think patients think about the idea in general?

What do you think the likely impact will be (do you have evidence for this)?
• On ED care delivery from perspective of ED department
• On primary care delivery across the community
• On patient care
• Have you considered unintended consequences (e.g. it will increase demand)
• On primary care delivery across the community
• On patient care
• Have you considered unintended consequences (e.g. it will increase demand)

Setting: Established Case Sites

Participants: Staff in ED/GPED/KI

What is your current role in the GPED?

What model of working with GPs/primary care operates in your ED currently?

Were you involved (and in what way) in the design or initial implementation of GPED?

- only if indicate were involved, ask planning/implementation questions

Planning/implementation stage:

What can you tell us about the initial process of design and implementation of this service

- Key staff involved
- Structural/organisational changes
- Decision making/service design
- Consultation with staff/patients/external bodies

What was expected to be achieved by the change?

What were the key barriers/facilitators?

What were the key issues for staff before the introduction?

What was the attitude/approach to change from management?

Impact:

How do you think the GPED model is working?

- Process of selecting patients to be seen by the GP/streaming/getting the 'right' patients
- Key advantages/disadvantages
- Any safety issues

How has it impacted on overall workings of the ED?

- Has there been any impact on performance (e.g. 4 hours, hospital admission rate)
- Resources

Do you think any improvements could be made to the GPED model (aware of different service configurations in other places)?

For peer review only - http://bmjopen.bmj.com/site/about/guidelines.xhtml

What feedback have you had from patients about the GPED model (are they satisfied etc)?

Do you think the availability of this GPED model is likely to change the way the public decide how, where and when to seek care?

For emergency care staff:

How has GPED impacted on your own everyday working?

- Clinically (type of patients/presenting conditions)
- Working relationships with other staff (e.g. the staff who select patients to be seen by GP, the GP staff)
- Service provided to patients
- Administratively/organizationally
- Any surprises

For general practice staff in GPED:

How is care organised within GP component of GPED?

How does practice within GPED compare to other services (GP practice, walk-in centres):

- Clinically (types of patients/presenting conditions)
- Patient 'outcomes' (e.g. referrals, requests for testing, transfer back to ED)
- Interaction with other professional groups within GP component/ED staff
- Workload
- Any surprises

Discussion around who is employer, professional indemnity, clinical supervision/support around clinical decision making in role as GP in ED

Do you feel you act differently as a practitioner following time in ED (probe – both back in primary care and over time within ED)

Satisfaction with role of GP in ED

- Met with expectations
- Plan to continue in role
- Career plans

How do you think patients have responded to the service?

For peer review only - http://bmjopen.bmj.com/site/about/guidelines.xhtml

GPED

- Why they came to AE rather than GP practice
- Satisfaction with GPED

Any other comments to add about GPED

For peer review only

For peer review only - http://bmjopen.bmj.com/site/about/guidelines.xhtml

GP ED

UWE Bristol University of the West of England

Setting: Existing Case Sites

Participants: Patients

What brought you to the ED on this occasion?

Tell us about what happened after you arrived?

- Who did you see first/what happened next
- Description of being selected to be seen by the GP

Did you know it was possible to be sent to a GP after coming to ED?

- Was this communicated to you
- Did you understand the process/reason you were selected for the GP
- How did you feel about being seen by a GP
- Have you any previous experience of this service (give example)

Explore reason behind attendance at ED for this consultation – why did they use ED over other potential services (walk-in centres, GP surgery)

- Knowledge of different ways to access health services and what they consider the 'appropriate' ways to use them

Would their experiences on this visit change their consultation choice in the future?

Explore awareness of increased demand on EDs/government funding made available to increase GPs in EDs

- Do they think GPs in ED good idea in principle
- What impact do they think it might have on reducing pressure on EDs
- Do they think it will change what patients do

How does practice within GPED compare to other GP services?

How satisfied are they with the visit?

- How long did you have to wait
- How satisfied are you with the outcome
- Can you think of any ways you could improve the service?
- Opportunity to provide feedback

Any other comments to add about GPED.

For peer review only - http://bmjopen.bmj.com/site/about/guidelines.xhtml

GPED

UWE Bristol University of the West of England

Setting: Prospective Case Sites

Timing: Before introduction of GPED

Participants: Staff in ED

Personal:

What is your current role in the ED?

Do you have a role in relation to the introduction of GPED? If so what is it?

GPED model:

Tell us what you understand about the GPED model that will be implemented in your department

Do you feel that many of the patients you see are 'inappropriate' for ED and should be in primary care (give examples)?

Are you aware of the background to the decision to introduce GPED:

- What it is hoped that GPED will achieve
- What discussions took place
- What options were considered
- What major factors impacted on decision making (if don't mention might want to prompt on waiting time, cost, numbers)
- Was there (describe) consultation process with staff/patients

How is it different from the model you have in place now (is it clearly distinct)

- Structural/organisational requirements for proposed model
- Training requirements
- Timetable for change (date)
- Knowledge/views on the process for selection of patients to be seen by the GP

What are your thoughts on the decision to fund these models of service delivery?

- Does the idea of GPs in ED make sense in general
- For your department
- Are you aware of other types of GPED models being implemented elsewhere

Do staff have a shared understanding of the purpose of the proposed model of GPED?

- Do staff feel they have had sufficient buy in

For peer review only - http://bmjopen.bmj.com/site/about/guidelines.xhtml

- What are your concerns (if any) regarding implementation
- Do you think there are any potential safety issues
- How supported do you feel by management going into the change

Expected impact:

What are your expectations of the impact of the new service on your own everyday working?

- Clinically (type of patients/presenting conditions)
- Working relationships with other staff (e.g. staff selecting patients to be seen by the GP, the GP staff)
- Administratively/organizationally
- For the service provided to patients

What you think the impact will be to your department on:

- Performance (4 hours, hospital admission rate)
- Resources
- How patients use the ED

What do you think will be the key barriers/facilitators to the introduction of GPEP?

What do you think would be deemed to be successful outcomes?

How do you think patients will respond to the new service (satisfaction, ability to feedback, change in behaviour)?

Any other comments to add about GPED

For peer review only

For peer review only - http://bmjopen.bmj.com/site/about/guidelines.xhtml

Setting: Prospective Case Sites

Timing: 'Before' introduction of GPED/early in implementation process

Participants: GPs

Personal:

What is your current role in the GPED?

What was your previous (or concurrent) role in primary care?

Did you have a role in relation to the introduction of GPED/how did you become aware of the new service model?

Explore decision around taking the role as GP in ED context

Discussion around who is employer, professional indemnity, clinical supervision/support around clinical decision making in role as GP in ED

GPED model:

Tell us what you understand about the GPED model that is being implemented

Are you aware of the background to the decision to introduce GPED:

- What it is hoped that GPED will achieve
- How the service came about
- Consultation process with CCG/other primary care forums

What are your thoughts on the decision to fund these models of service delivery?

- Does the idea of GPs in ED make sense in general
- Aware of other types of GPED models being implemented elsewhere

Do staff (from GP component of service) have a shared understanding of the purpose of the proposed model of GPED?

- Do staff feel they have had sufficient buy in
- What are your concerns (if any) regarding implementation
- Do you think there are any potential safety issues
- How supported do you feel by management going into the change

For peer review only - http://bmjopen.bmj.com/site/about/guidelines.xhtml

Expected impact:

What are your expectations of the impact of the new service on your own everyday working?

- Clinically (type of patients/presenting conditions)
- Working relationships with other staff (e.g. staff selecting patients to be seen by the GP, the ED staff)
- Administratively/organizationally
- For the service provided to patients

What you think the impact will be to your ED department on:

- Performance (4 hours, hospital admission rate)
- Resources
- How patients use the ED

What do you think will be the key barriers/facilitators to the introduction of GPED?

What do you think would be deemed to be successful outcomes?

How do you think patients will respond to the new service (satisfaction, ability to feedback, change in behaviour)?

Any other comments to add about GPED

For peer review only - http://bmjopen.bmj.com/site/about/guidelines.xhtml

Setting: Prospective Case Sites

Timing: Before introduction of GPED

Participants: Key informants

Personal:

What is your current role in the ED?

What is your role in relation to the introduction of GPED?

ED context:

What model of working with GPs/primary care operates in your ED currently (if any)?

GPED model:

Tell us about the GPED model you are planning to implement

Can you tell us the background to that decision:

- What you are hoping to achieve
- What discussions took place
- What options were considered
- What major factors impacted on decision making (if don't mention might want to prompt on waiting time, cost, numbers)
- Describe the process of consultation (with external bodies e.g. CCG/with internal staff/with patients (or patient reps)

How is it different from the model you have in place now (is it clearly distinct)

- Structural requirements for proposed model
- Organisational requirements for proposed model
- How will changes (if any) be achieved
- Timetable for change (date)

What are your thoughts on the decision to fund these models of service delivery

- does the idea of GPs in ED make sense in general

Do you think this model makes sense/is the right thing for your department?

Do you think staff value the proposed model of service provision?

For peer review only - http://bmjopen.bmj.com/site/about/guidelines.xhtml

- Do staff have a shared understanding of the purpose of the proposed model of GPED
- Do staff feel they have had sufficient buy in
- What are the concerns (if any) raised by staff regarding implementation
- Can you foresee any potential safety issues

How will you select patients to be seen by the GP and ensure these are the 'right' patients?

- How will you draw the distinction between GP and ED care

Expected impact:

What do you think the impact will be to your department on:

- Performance (4 hours, hospital admission rate)
- Staff (which staff in particular, in what ways)
- Division of labour
- Interaction between different professional groups
- Resources

What impact do you expect GPED to have on patient care?

- Do you think patients will be satisfied with the model
- Do you have a mechanism to collect and/or respond to feedback from patients

Will staff require additional training before implementation

- Which staff and what training in planned/available

How will you judge the success/impact of the new model of service delivery:

- What data might be available for research purposes
- Mechanism for staff feedback about the intervention
- Can the intervention be adapted on the basis of experience
- Patient outcomes

What impact do you think GPED will have on how the public access ED/primary care services?

- How does it sit with other services including walk-in centres, GP practices

Any other comments to add about GPED

For peer review only - http://bmjopen.bmj.com/site/about/guidelines.xhtml

GP+ED

UWE Bristol University of the West of England

Setting: Prospective Case Sites

Timing: Before GPED

Participants: Patients

What brought you to the ED on this occasion?

Tell us about what happened after you arrived?

- Who did you see first/what happened next
- Description of triage process

Explore reason behind attendance at ED for this consultation – why did they use ED over other potential services (walk-in centres, GP surgery)

- Knowledge of different ways to access health services and what they consider the 'appropriate' ways to use them

Would their experiences on this visit change their consultation choice in the future?

Explore awareness of increased demand on EDs/government funding made available to increase GPs in EDs

- Do they think GPs in ED good idea in principle
- What impact do they think it might have on reducing pressure on EDs

Briefly describe model being proposed and seek comments on that

- What features would make that a good service for patients
- Can see any advantages/disadvantages
- How might they have felt about seeing a GP on this visit

Any other comments to add about GPED.

For peer review only - http://bmjopen.bmj.com/site/about/guidelines.xhtml

Standards for Reporting Qualitative Research (SRQR)*

http://www.equator-network.org/reporting-guidelines/srqr/

Page/line no(s).

Title and abstract

**Title** - Concise description of the nature and topic of the study Identifying the study as qualitative or indicating the approach (e.g., ethnography, grounded theory) or data collection methods (e.g., interview, focus group) is recommended	1 (1-2)
**Abstract** - Summary of key elements of the study using the abstract format of the intended publication; typically includes background, purpose, methods, results, and conclusions	2 (34-58)

Introduction

**Problem formulation** - Description and significance of the problem/phenomenon studied; review of relevant theory and empirical work; problem statement	3-5 (74-118)
**Purpose or research question** - Purpose of the study and specific objectives or questions	4 / (112-118)

Methods

**Qualitative approach and research paradigm** - Qualitative approach (e.g., ethnography, grounded theory, case study, phenomenology, narrative research) and guiding theory if appropriate; identifying the research paradigm (e.g., postpositivist, constructivist/ interpretivist) is also recommended; rationale**	5(121-137)<br>6(140-147)
**Researcher characteristics and reflexivity** - Researchers' characteristics that may influence the research, including personal attributes, qualifications/experience, relationship with participants, assumptions, and/or presuppositions; potential or actual interaction between researchers' characteristics and the research questions, approach, methods, results, and/or transferability	7 (164-167)
**Context** - Setting/site and salient contextual factors; rationale**	6 table 1 142-162
**Sampling strategy** - How and why research participants, documents, or events were selected; criteria for deciding when no further sampling was necessary (e.g., sampling saturation); rationale**	6 (142-147); 21 (435-439)
**Ethical issues pertaining to human subjects** - Documentation of approval by an appropriate ethics review board and participant consent, or explanation for lack thereof; other confidentiality and data security issues	5 (box 1) 23 (477-481)
**Data collection methods** - Types of data collected; details of data collection procedures including (as appropriate) start and stop dates of data collection and analysis, iterative process, triangulation of sources/methods, and modification of procedures in response to evolving study findings; rationale**	6 (40-141);6-7 (148-162)

For peer review only

**Data collection instruments and technologies** - Description of instruments (e.g., interview guides, questionnaires) and devices (e.g., audio recorders) used for data collection; if/how the instrument(s) changed over the course of the study	6-7 (140-162)Topic guides attached as supplementary files.
**Units of study** - Number and relevant characteristics of participants, documents, or events included in the study; level of participation (could be reported in results)	6 (140-151)
**Data processing** - Methods for processing data prior to and during analysis, including transcription, data entry, data management and security, verification of data integrity, data coding, and anonymization/de-identification of excerpts	7 (164-176)
**Data analysis** - Process by which inferences, themes, etc., were identified and developed, including the researchers involved in data analysis; usually references a specific paradigm or approach; rationale**	7(154-176)
**Techniques to enhance trustworthiness** - Techniques to enhance trustworthiness and credibility of data analysis (e.g., member checking, audit trail, triangulation); rationale**	6-7(164-188)

Results/findings

**Synthesis and interpretation** - Main findings (e.g., interpretations, inferences, and themes); might include development of a theory or model, or integration with prior research or theory	Throughout 8-19)(189-368)
**Links to empirical data** - Evidence (e.g., quotes, field notes, text excerpts, photographs) to substantiate analytic findings	Throughout 9-19)(189-368)

Discussion

**Integration with prior work, implications, transferability, and contribution(s) to the field -** Short summary of main findings; explanation of how findings and conclusions connect to, support, elaborate on, or challenge conclusions of earlier scholarship; discussion of scope of application/generalizability; identification of unique contribution(s) to scholarship in a discipline or field	19-22 (369-460)
**Limitations** - Trustworthiness and limitations of findings	3 (71-72) 21 (430-449)

Other

**Conflicts of interest** - Potential sources of influence or perceived influence on study conduct and conclusions; how these were managed	22 (473-475)
**Funding** - Sources of funding and other support; role of funders in data collection, interpretation, and reporting	5(137)22 (465-467)

*The authors created the SRQR by searching the literature to identify guidelines, reporting standards, and critical appraisal criteria for qualitative research; reviewing the reference lists of retrieved sources; and contacting experts to gain feedback. The SRQR aims to improve the transparency of all aspects of qualitative research by providing clear standards for reporting qualitative research.

For peer review only
For peer review only - http://bmjopen.bmj.com/site/about/guidelines.xhtml

**The rationale should briefly discuss the justification for choosing that theory, approach, method, or technique rather than other options available, the assumptions and limitations implicit in those choices, and how those choices influence study conclusions and transferability. As appropriate, the rationale for several items might be discussed together.

Reference:
O'Brien BC, Harris IB, Beckman TJ, Reed DA, Cook DA. **Standards for reporting qualitative research: a synthesis of recommendations.** *Academic Medicine*, Vol. 89, No. 9 / Sept 2014 DOI: 10.1097/ACM.0000000000000388

For peer review only

For peer review only - http://bmjopen.bmj.com/site/about/guidelines.xhtml
